# Deletion of the polyketide synthase‐encoding gene *pks1* prevents melanization in the extremophilic fungus *Cryomyces antarcticus*

**DOI:** 10.1002/iub.2895

**Published:** 2024-07-16

**Authors:** Ilaria Catanzaro, Ruben Gerrits, Ines Feldmann, Anna A. Gorbushina, Silvano Onofri, Julia Schumacher

**Affiliations:** ^1^ Department Materials and the Environment Bundesanstalt für Materialforschung und ‐prüfung (BAM) Berlin Germany; ^2^ Department of Ecological and Biological Sciences (DEB) Università degli Studi della Tuscia Viterbo Italy; ^3^ Department of Biology, Chemistry, Pharmacy Freie Universität Berlin Berlin Germany

**Keywords:** astrobiology, black fungi, carotenoids, CRISPR/Cas9, cryptoendolithism, DHN melanin, stress tolerance

## Abstract

*Cryomyces antarcticus*, a melanized cryptoendolithic fungus endemic to Antarctica, can tolerate environmental conditions as severe as those in space. Particularly, its ability to withstand ionizing radiation has been attributed to the presence of thick and highly melanized cell walls, which—according to a previous investigation—may contain both 1,8‐dihydroxynaphthalene (DHN) and L‐3,4 dihydroxyphenylalanine (L‐DOPA) melanin. The genes putatively involved in the synthesis of DHN melanin were identified in the genome of *C. antarcticus.* Most important is *capks1* encoding a non‐reducing polyketide synthase (PKS) and being the ortholog of the functionally characterized *kppks1* from the rock‐inhabiting fungus *Knufia petricola*. The co‐expression of CaPKS1 or KpPKS1 with a 4′‐phosphopantetheinyl transferase in *Saccharomyces cerevisiae* resulted in the formation of a yellowish pigment, suggesting that CaPKS1 is the enzyme providing the precursor for DHN melanin. To dissect the composition and function of the melanin layer in the outer cell wall of *C. antarcticus*, non‐melanized mutants were generated by CRISPR/Cas9‐mediated genome editing. Notwithstanding its slow growth (up to months), three independent non‐melanized Δ*capks1* mutants were obtained. The mutants exhibited growth similar to the wild type and a light pinkish pigmentation, which is presumably due to carotenoids. Interestingly, visible light had an adverse effect on growth of both melanized wild‐type and non‐melanized Δ*capks1* strains. Further evidence that light can pass the melanized cell walls derives from a mutant expressing a H2B‐GFP fusion protein, which can be detected by fluorescence microscopy. In conclusion, the study reports on the first genetic manipulation of *C. antarcticus*, resulting in non‐melanized mutants and demonstrating that the melanin is rather of the DHN type. These mutants will allow to elucidate the relevance of melanization for surviving extreme conditions found in the natural habitat as well as in space.

## 1 INTRODUCTION

*Cryomyces antarcticus* (class Dothideomycetes) is a cryptoendolithic black fungus endemic to Antarctica.^1^, ^2^ In addition to displaying morpho‐physiological traits shared among microcolonial fungi, *C. antarcticus* exhibits an extremely slow meristematic growth—an isodiametric cellular expansion optimizing the surface/volume ratio and a stable character of psychrophilic fungi even under optimal culture conditions.^3^ Isolated from sandstone sampled at Linnaeus Terrace (McMurdo Dry Valleys, Southern Victoria Land), the fungus has been preserved in the National Antarctic Museum—Culture Collection of Fungi from Extreme Environments (MNA‐CCFEE) at the University of Tuscia in Viterbo, Italy.^4^

Since the discovery of black fungi within cryptoendolithic microbial communities in Antarctic rocks,^5^ large attention has been directed towards the fungi due to their distinctive adaptations to extreme environmental conditions.^6^ *C. antarcticus* strain MNA‐CCFEE 515 has been particularly studied under a variety of conditions, from those typical of its habitat (e.g., severe thermal fluctuations, intense UV‐B irradiation), to the more exotic found in space or extraplanetary environments (e.g., vacuum, ionizing radiation).^4^ Its remarkable ability to thrive in such diverse extreme conditions has been ascribed to its unique morpho‐physiological adaptations,^7^ specifically melanin cross‐linked to the thick cell walls,^8^ rather than to a robust DNA repair system.^9^

Indeed, fungal melanins contribute to the structural integrity of the cell wall and protection against cellular stress.^10^ These heterogenic macromolecules result from the oxidative polymerization of phenolic/indolic precursors and are known for their negatively charge and hydrophobic nature.^11^ Fungi may employ two distinct biosynthetic approaches: the 1,8‐dihydroxynaphthalene (DHN) pathway, which entails a polyketide synthase (PKS) catalyzing the de novo synthesis of phenolic substrates for DHN melanin polymerization, and the L‐3,4 dihydroxyphenylalanine (L‐DOPA) pathway, which converts tyrosine or L‐DOPA through tyrosinase and laccase activity into dopaquinone for spontaneous DOPA melanin polymerization.^12^ The former is the prevalent strategy found in ascomycetes, while the latter appears to be more common in basidiomycetes and human pathogens.^13^, ^14^ An additional dark pigment found in fungi, the water‐soluble pyomelanin, lacks its own biosynthesis pathway and derives from polymerization of an L‐tyrosine degradation product.^15^, ^16^ Though the tyrosine from primary metabolism can be used for both DOPA melanin and pyomelanin synthesis, fungi usually produce these pigments only when the precursors are exogenously supplied.^17^

Investigations into the melanin of *C. antarcticus* have primarily relied on chemical analytical methods,^18^, ^19^ without a clear assessment of the genetic basis behind the biosynthesis process. Our objective is to fill this research gap by adopting a genetic engineering approach in *C. antarcticus*. This methodology offers more consistent and reproducible results compared with chemical inhibitors and other destructive techniques,^20^ and allows to study the functions *in vivo*. However, the development of effective molecular tools for meristematic black fungi has traditionally faced challenges compared with filamentous fungi. Their slow growth and heavy melanization make them difficult to manipulate genetically. The development of the CRISPR (Clustered Regularly Interspaced Short Palindromic Repeats)/Cas9 (CRISPR‐associated protein 9) technology represents the opportunity to establish techniques for targeted genome editing, even in hard‐to‐reach fungi, as it was recently demonstrated for the rock‐inhabiting fungus *Knufia petricola* (Eurotiomycetes/Chaetothyriales).^21^ With a relatively faster growth compared with *C. antarcticus*, this fungus displays morpho‐physiological traits typical of black fungi.^22^, ^23^, ^24^ The production of DHN melanin, carotenoids, mycosporines, and extracellular polysaccharides (EPS) has been well‐characterized.^25^, ^26^, ^27^, ^28^ Originally isolated from a marble surface in Athens, Greece,^29^ the strain A95 (CBS 123872) serves as model for exploring the lifestyle and physiology of material‐colonizing black fungi.^30^ The annotated genome sequence and toolkits for genetic engineering and comparative phenotyping^21^, ^31^ supply essential resources for targeted gene knock‐out and knock‐in approaches and the functional characterization of the generated strains. For instance, *K. petricola* mutants deficient in the synthesis of DHN melanin, carotenoids, or both pigments are used to elucidate their roles in traits such as the dissolution of minerals and penetration of carbonate substrates.^32^, ^33^, ^34^

In this work, we made *C. antarcticus* the first Antarctic fungus to undergo genetic engineering, for facilitating the study of the role of its pigments in withstanding extreme natural and extraterrestrial conditions in future investigations. The availability of CRISPR/Cas9 editing techniques, along with previous experiences in transforming *K. petricola*, facilitated the establishment of transformation protocols for *C. antarcticus*. Our approach involved the targeting of the key enzyme‐encoding gene for DHN melanogenesis (*capks1*) and the putative carotenogenic genes (*caphs1*, *caphd1*). It resulted in the successful generation of DHN melanin‐ and carotenoid‐deficient *C. antarcticus* mutants, demonstrating the feasibility of genetically manipulating less amenable fungi.

## 2 EXPERIMENTAL PROCEDURES

### 2.1 Cultivation of *C. antarcticus*

*C. antarcticus* and *K. petricola* strains listed in Table 1 were cultivated on the standard media used for maintenance of *K. petricola* strains, that is, on malt extract broth/agar (MEB/MEA: 2.0% malt extract [Carl Roth GmbH + Co. KG], 0.1% peptone from casein [Carl Roth GmbH + Co. KG], 2.0% D‐glucose, 2.0% kobe agar [AppliChem GmbH]), a synthetic‐defined nitrate–glucose medium (SDNG: 0.17% Difco Yeast Nitrogen Base without Amino Acids and Ammonium Sulfate [BD Biosciences], 0.3% NaNO_3_, 2.0% D‐glucose, 2.0% kobe agar), or an additional malt extract agar (MEAV: 3.0% malt extract [Carl Roth GmbH + Co. KG], 1.5% bacteriological agar [AppliChem GmbH]). MEA and SDNG media were supplemented with the selective agents hygromycin B (HYG; AppliChem), nourseothricin (NTC; Werner BioAgents GmbH), geneticin (G418; Sigma‐Aldrich), or glufosinate ammonium (GFS; ChemPur) as specified. *C. antarcticus* biomass from 8‐week‐old MEA cultures were homogenized using a Retsch mixer mill (5 min at 30 Hz). Titers of colony‐forming units (CFU) were determined using a Thoma cell counting chamber and adjusted with 1x phosphate‐buffered saline (PBS) to 5 × 10^6^ cells/ml for growth assays and 1 × 10^6^ cells/ml for drop assays. For growth assays, serial dilutions down to 5 × 10^3^ cells/ml were prepared, and 200 μL (1 × 10^6^ to 1 × 10^3^ cells) were evenly distributed with 10 glass beads (3–5 mm) on agar, while 100 μL of the highest diluted cell suspension (5 × 10^2^ cells) were used as inoculum per quarter with four‐compartment Petri dishes. For drop assays, serial dilutions down to 1 × 10^3^ cells/ml were prepared, and 10 μL (1 × 10^4^ to 1 × 10^1^ cells) were dropped in a grid pattern onto agar. For liquid cultures, 20 mL of MEB were inoculated with 200 μL of homogenized cell suspensions. *K. petricola* strains on solidified media were incubated at 25°C in the dark, and *C. antarcticus* strains at 15°C in constant darkness (DD) or in a 12 h light/12 h dark (LD) cycle (Philips F32T8/TL741 cool white, 118 μmol photons/m^2^/s).

**TABLE 1:** Fungal strains used in this study.

Strain name	BAM ID	Genotype	Reference
*C. antarcticus* MNA‐CCFEE 515		WT	1
Δ*capks1*	CA‐0002	515, Δ*pks1* [(T*niaD*::*hph*::P*trpC*)]	This study
Δ*caphs1‐phd1*	CA‐0017	515, Δ*phs1*‐*phd1* [(T*niaD*::*hph*::P*trpC*)]	This study
*h2b‐gfp*	CA‐0021	515, Δ*phs1*‐*phd1* [(T*niaD*::*hph*::P*trpC*)–(P*oliC*::*h2b‐gfp*::T*gluc*)]	This study
*K. petricola* A95		WT	23
Δ*kpsdh1*	KP‐0053	A95, Δ*sdh1* [(T*niaD*::*nat1*::P*trpC*)]	21
Δ*kppks1*	KP‐0033	A95, Δ*pks1* [(T*niaD*::*nat1*::P*trpC*)]	21
*kppks1* ^COMiL^	KP‐0505	A95, P*pks1*::[(*pks1*::T*gluc*)‐(T*niaD*::*nat1*::P*trpC*)]	This study
*S. cerevisiae* FY834		*MAT*α *his3‐*Δ*200 ura3‐52 leu2‐*Δ*1 lys2‐*Δ*202 trp1‐*Δ*63*	35
Control	SC‐0271	FY834, p425 GAL1, p426 GAL1	This study
*kpppt1*	SC‐0272	FY834, pLEU‐*kpppt1*, p426 GAL1	This study
*kppks1*	SC‐0273	FY834, p425 GAL1, pURA‐*kppks1*	This study
*kpppt1 + kppks1*	SC‐0274	FY834, pLEU‐*kpppt1*, pURA‐*kppks1*	This study
*capks1*	SC‐0277	FY834, p425 GAL1, pURA‐*capks1*	This study
*kpppt1 + capks1*	SC‐0278	FY834, pLEU‐*kpppt1*, pURA‐*capks1*	This study

### 2.2 Bioinformatics analyses

*C. antarcticus* nucleotide and protein sequences of putative melanogenic (Figure S1) and carotenogenic genes listed in Table S1 were extracted from the *C. antarcticus* CBS 116301 v3.0 database (https://mycocosm.jgi.doe.gov/Cryan3) at the Joint Genome Institute. Accession numbers of protein sequences used for sequence comparisons are listed in Table S2. For muscle alignments, phylogenetic trees, and the identification of CRISPR sites, the corresponding tools of Geneious Prime 2023.2.1 (Biomatters Ltd.) were used. Conserved protein domains were identified by InterPro (https://www.ebi.ac.uk/interpro).^36^ Plasmid maps were generated with SnapGene 4.0.8 (GSL Biotech LLC).

### 2.3 Standard molecular methods

Genomic DNA from *C. antarcticus* was extracted as described previously for *K. petricola*.^21^ DNA and the 1 kb Plus DNA Ladder (New England Biolabs, NEB), mixed with Midori Green Direct (Biozym Scientific GmbH), were separated in 1% agarose gels in a Mupid exU gel electrophoresis chamber filled with 0.5% tris‐acetate‐EDTA (TAE) buffer and visualized by using a ChemiDoc XRS+ Imager equipped with the Image Lab 6.0.1 software (Bio‐Rad Laboratories Inc.). DNA was amplified with the Q5 High‐Fidelity DNA Polymerase (New England Biolabs, NEB) for cloning and transformation and with the *Taq* DNA Polymerase (NEB) for diagnostic applications. Primers listed in Table S3 were obtained from Eurofins Genomics. Plasmids listed in Table S4 were assembled *in vivo*, that is, in *Saccharomyces cerevisiae* FY843,^37^ or *in vitro* by using the NEBuilder HiFi DNA Assembly Master Mix (NEB). Plasmid DNA from *Escherichia coli* and *S. cerevisiae* was extracted with the Monarch Plasmid Miniprep Kit (NEB). Larger amounts of plasmid DNA from *E. coli* were extracted with the NucleoBond Xtra Midi Kit (Macherey‐Nagel). Sanger sequencing of plasmid DNA was accomplished at Eurofins Genomics.

### 2.4 Gene expression in *S. cerevisiae*

Vectors for galactose‐inducible expression of *kpppt1* (GenBank: MT859418.1), *kppks1* (GenBank: PP374627.1), and *capks1* (Cryan3 scaffold_6:122131–129,243) in *S. cerevisiae* (pLEU‐*kpppt1*, pURA‐*kppks1*, pURA‐*capks1*) were assembled *in vivo* using p425 GAL or p426 GAL1^38^ as entry plasmid, as described in Figure S2A and Table S4. *S. cerevisiae* FY834 was transformed using the LiAc/single‐stranded carrier DNA/PEG method from Gietz and Schiestl.^39^ Plasmid‐propagating strains (Table 1) were maintained on glucose‐containing SD medium lacking the respective nutrients. SD/GLU‐HLWU medium (2.0% D‐glucose, 0.17% Difco Yeast Nitrogen Base without Amino Acids and Ammonium Sulfate [BD Biosciences], 0.5% [NH_4_]_2_SO_4_, 0.06% drop‐out supplement –HIS/–LEU/–TRP/–URA [Takara Bio Inc.], 2.0% kobe agar; pH 5.8) was supplemented with 10 mg/L of histidine, 50 mg/L of tryptophane, 100 mg/L of leucine, and/or 50 mg/L of uracil. For induction of gene expression, the strains were cultivated on SD/GAL‐LU agar containing 4.0% D‐galactose instead of D‐glucose as carbon source at 30°C in darkness.

### 2.5 Genetic manipulation of *C. antarcticus*

Procedures for editing the genome of *C. antarcticus* were adopted from those established in *K. petricola* ^21^, ^31^, ^40^ (Figure S3). For introducing double strand breaks (DSBs) in the *genes of interest* (*goi*), target‐specific sgRNA and Cas9 were transiently expressed from a circular plasmid. Plasmids coding for Cas9 and one or two sgRNA (pAMA/tRNA‐*capks1* ^PS1^‐*capks1* ^PS4^, pAMA/tRNA‐*caphs1* ^PS1^ *‐caphd1* ^PS1^, pAMA/tRNA‐*hph* ^PS1^, and pAMA/tRNA‐*nat1* ^PS1^) were cloned by assembly of sgRNA/tRNA fragments amplified from pFC902^41^ in *Pac*I‐digested pFC332^42^ as described in detail in Table S4. Linear donor DNA for replacing genes of interest was generated by amplification or by digestion of plasmid DNA. Replacement fragments with 75‐bp‐long homologous sequences were amplified from pNDH‐OGG, pNDN‐OGG,^37^ or pNDH‐ONGG (Erdmann et al., under review) with primers *goi*‐SH5F and *goi*‐SH3R/T*gluc*‐*goi*‐SH3R as exemplarily shown in Figure S4. For reintroduction of genes in replacement mutants into their native genomic loci, the expression constructs with long homologous sequences were isolated with *Swa*I from cloned pN‐*capks1* ^COMIL^ and pH‐*kppks1* ^COMIL^ (Tables S4 and S5, Figure S5). Protoplasts of *C. antarcticus* strains were generated by enzymatic digestion of the cell walls (Figure S3). For that, 200 to 300 mg of biomass from a 4‐week‐old MEB culture of the *C. antarcticus* strain of interest was washed twice with protoplast buffer (KPB) and then resuspended in 20 mL of protoplast buffer (KPB) containing 40 mg/mL of VinoTaste Pro (Novozymes) and 1 mg/mL of Yatalase (Takara Bio Inc.). Cell wall lysis was performed at 27°C and 80 rpm overnight. Protoplasts were collected using a 15‐μm cell strainer (pluriSelect), followed by centrifugation at 1000 × g and 4°C for 5 min. Protoplasts were washed twice with transformation buffer (KTB) and resuspended in the same buffer at a final concentration of ≥3.5 × 10^6^ protoplasts/ml. 1–4 × 10^6^ protoplasts in volumes of 100 to 200 μL were mixed with 40 μg of plasmid DNA and 15–30 μL of donor DNA (resistance cassettes) and transformed in a 1:2:15 volume ratio with 24% PEG 6000 and liquid transformation medium (KTM). Transformation reactions were gently distributed over 20 mL of osmotically stabilized MEA (MEAS) for obtaining ~10^6^ protoplasts per Petri dish. After incubation for 24 h at 15°C, the plates were overlaid with 5 mL of KTM top agar supplemented with either 125 μg/mL of HYG (final conc. 25 μg/mL) or 25 μg/mL of NTC (final conc. 5 μg/mL). Putative resistant transformants were transferred to MEA containing equal HYG or NTC concentrations after 2 to 4 months. Growing strains were repeatedly transferred to fresh selective MEA, before DNA from chosen transformants was extracted. Homologous recombination (HR) events resulting in the replacement of the gene of interest by a resistance cassette were detected by diagnostic PCR using primers binding in the used resistance cassette (T*niaD*‐hiF, *hph*‐hiF, *hph*‐hiR, *ogfp*‐sF1) and the up‐ and downstream of the targeted gene (*goi*‐hi5F, *goi*‐hi3R) as specified in Figures S4–S6.

### 2.6 Microscopic analyses

For scanning electron microscopy (SEM), samples were cut out of solid agar medium covered with either the *C. antarcticus* wild‐type or the Δ*capks1* mutant (two for each) and prepared according to Spurr.^43^ This protocol included a fixation with 2.5% glutaraldehyde, a wash with PBS, and a dehydration using an ethanol dilution series with gradually increasing concentration (i.e., 30% to 100%). Afterwards, samples were dried via critical point drying (Leica EM CPD300) and fixed onto SEM sample holders using adhesive carbon tape. After sputter coating with a 30 nm conducting gold layer (EM ACE600), they were analyzed by SEM (FEI XL30 ESEM) at the electron microscopy center at BAM. SEM images were acquired with a secondary electron detector at an accelerating voltage of 20 kV. For transmission electron microscopy (TEM), *C. antarcticus* wild‐type and Δ*capks1* colonies were collected in quadruplicates from 8‐week‐old MEA cultures and prepared according to an optimized protocol.^44^ Sections obtained using the Reichert Ultracut ultramicrotome were stained with uranyl acetate and lead citrate, and image acquisition was performed at the Great Equipment Center, section of Electron Microscopy of the University of Tuscia, as previously reported.^45^ For fluorescence microscopy, cells were homogenized using a Retsch mixer mill (5 min at 30 Hz), washed twice with PBS and transferred to microscope slides. For staining the nuclei, cells were resuspended in PBS containing 20 μg/mL of DAPI (4′,6‐diamidino‐2‐phenylindole) (Sigma‐Aldrich), incubated for 3 min in the dark at room temperature and washed twice with PBS. DAPI and GFP fluorescence was detected with a Leica SP8 confocal laser scanning microscope using a 100x objective with immersion oil. GFP was exited at 485 nm, and the emission was recorded at 530/40 nm using a PMT detector. DAPI was exited at 405 nm, and the emission was detected at 460/40 nm using a HyD detector.

## 3 RESULTS AND DISCUSSION

### 3.1 Identification of the putative DHN melanogenic genes in *C. antarcticus*

DHN melanin is produced by many ascomycetes. Filamentous fungi often incorporate DHN melanin in the cell walls of their reproductive structures such as conidiophores and conidia, fruiting bodies, and sexual spores or sclerotia, while the vegetive mycelia are often not melanized. For example, DHN melanogenesis, its regulation, and functions have been studied in detail in *Aspergillus fumigatus*,^46^ *Alternaria alternata*,^47^ *Neurospora crassa*,^48^ and *Botrytis cinerea*.^49^ By contrast, black fungi that reproduce asexually by budding or meristematic growth only produce the dark pigment constitutively.^50^ The biosynthetic routes for the phenolic precursor DHN are rather conserved (Figure S1), though the organization and regulation of the involved genes differ among species. Not yet well understood is how the DHN is polymerized outside the cell and which chemical structure or modifications the final DHN polymers have. As DHN can be cross‐linked with other metabolites and components of the cell wall including chitin, glucan, and glycoproteins, the final polymer may have heterogenous compositions.

**FIGURE 1:**
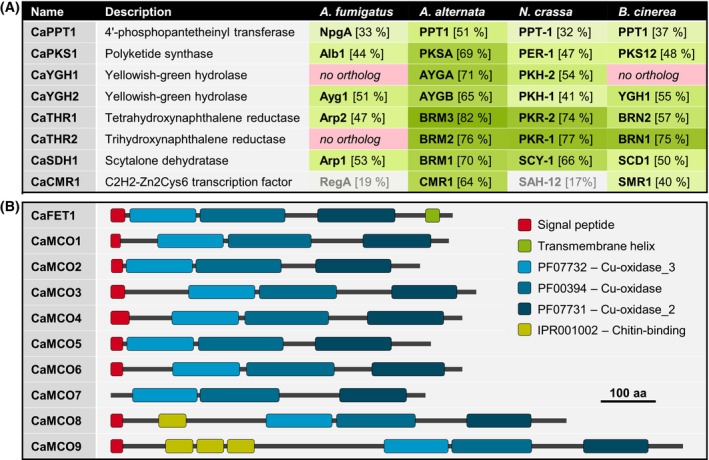
Putative DHN melanogenic enzymes of *C. antarcticus*. (A). Enzymes putatively involved in the formation of DHN. Orthologs from *Aspergillus fumigatus* (Eurotiomycetes), *Alternaria alternata* (Dothideomycetes), *Neurospora crassa* (Sordariomycetes), and *Botrytis cinerea* (Leotiomycetes) (% amino acid identity with *C. antarcticus* proteins in brackets) are shown. See Tables S1 and S2 for accession numbers. (B). Multicopper oxidases (MCOs) that might be involved in polymerization of DHN. Proteins shown were identified by the presence of the three conserved Cu‐oxidase domains. FET1 contains a C‐terminal transmembrane helix typical for ferroxidases. MCOs with an N‐terminal signal peptide might be secreted.

**FIGURE 2:**
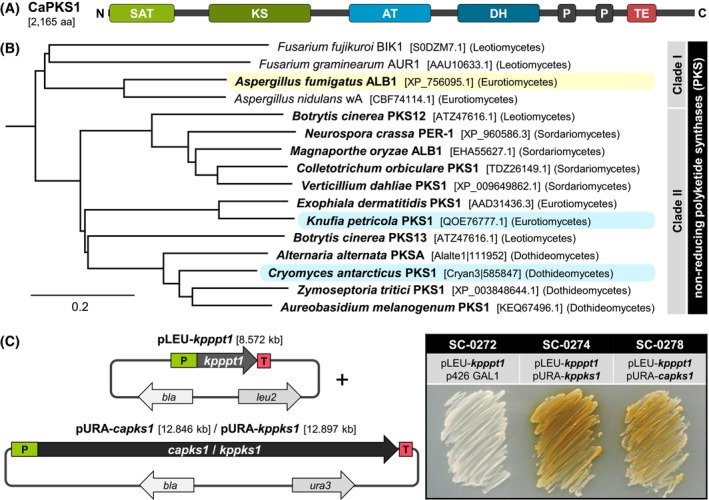
The polyketide synthase PKS1 of *C. antarcticus*. (A). Domain organization of CaPKS1. SAT—starter unit: ACP transacylase (PF16073); KS—β‐ketoacyl synthase (PF00109, PF02801); AT—acyl transferase (PF00698); DH—dehydratase (PF14765); P—phosphopantetheine attachment site (PF00550); TE—thioesterase (PF00975). (B). CaPKS1 belongs to clade II of non‐reducing PKS. Species indicated in bold produce DHN melanin. ALB1, the only melanin‐producing PKS in clade I is highlighted yellow; the PKSs of rock‐inhabiting species in clade II are highlighted blue. (C). Expression of CaPKS1 in *Saccharomyces cerevisiae* results in a yellowish pigment. *S. cerevisiae* strain FY834 was transformed with two plasmids for the expression of one or two genes from *K. petricola* (*kpppt1* encodes a phosphopantetheinyl transferase [PPT], *kppks1* the PKS for DHN melanogenesis) or *C. antarcticus* (*capks1*). P—promoter; T—terminator. Plasmid‐propagating *S. cerevisiae* strains were cultivated for 3 days at 30°C on SD/GAL‐LU agar for induction of gene expression. For further details and controls see Table 1 and Figure S2.

To identify the putative DHN melanogenic genes in the genome of *C. antarcticus* (Table S1), BlastP searches were conducted using the amino acid sequences of the *A. fumigatus* melanogenic enzymes as queries (Table S2). Genes for all necessary enzymatic steps were found (Figure 1A). This includes a polyketide synthase (PKS1) with a characteristic domains structure (Figure 2A) and a phosphopantetheinyl transferase (PPT1), both needed for the formation of the phenolic backbone, and enzymes for converting the intermediates to DHN (YGH1, YGH2, THR1, THR2, SDH1) (Figure S1). The sequences of the *C. antarcticus* enzymes were compared with orthologs from DHN melanin‐producing fungi of other classes. As expected, the *C. antarcticus* sequences display the highest amino acid identities (51%–82%) with those of *A. alternata* (Dothideomycetes), the closest relative among the four fungi compared. All fungi examined exhibit two THR reductases, except for *A. fumigatus*, which possess only one carrying out both reduction steps in the DHN pathway.^46^ According to the findings in other fungi where both THN reductases have a redundant function but prefer T4HN or T3HN as substrate,^51^ *C. antarcticus* THR1 and THR2 may also be redundant exhibiting preference for T4HN and T3HN, respectively.

Best known for their relevance for DHN polymerization are the two enzymes ABR1 and ABR2, which are encoded by genes in the DHN melanin cluster of *A. fumigatus*. Both are multicopper oxidases (MCO), whose deletions cause altered pigmentation.^46^ BlastP searches in the database of annotated *C. antarcticus* proteins revealed 10 proteins (Figure 1B) containing the three characteristic Cu‐oxidase domains and the conserved patterns for coordinating the four copper atoms which are characteristic for fungal LCCs,^52^ namely HxH (L1), HxH (L2), HxxHxH (L3), and HCHxxxHxxxG[M/F/L] (L4). Interestingly, CaMCO8 and CaMCO9 have extended N‐terminal regions bearing one and three predicted chitin‐binding domains, respectively. N‐terminal signal peptides for secretion were predicted for all *C. antarcticus* proteins except for CaMCO7. Because of similarity to *A. fumigatus* FetC (62% aa identity), the characteristic C‐terminal transmembrane domain and physical linkage of *cafet1* with an iron permease‐encoding gene, CaFET1 was identified as a ferroxidase putatively involved in reductive iron assimilation. In sum, the *C. antarcticus* genome bears all genes for the synthesis of DHN and its extracellular polymerization by secreted MCOs.

### 3.2 *C. antarcticus* PKS1 produces a yellowish pigment when expressed in *S. cerevisiae*

Fungal non‐reducing PKSs produce precursors for differently colored pigments by using acetyl‐coA and malonyl‐coA as substrates.^53^ The precursors themselves may be already colored. Clade I enzymes such as *A. fumigatus* ALB1, *A. nidulans* wA, and *Fusarium graminearum* PKS12 release the yellowish heptaketide YWA1, which is then modified in different pathways (Figure S1). In *A. fumigatus*, YWA1 is deacetylated by the hydrolase AYG1 to yield the pentaketide T4HN, with is further converted to DHN and DHN melanin.^54^ In *A. nidulans*, YWA1 is oxidized in the conidia by the laccase yA to a greenish pigment.^55^ In *F. graminearum*, YWA1 is converted in several steps to the red pigment aurofusarin.^56^ Clade II PKSs produce different polyketides for DHN melanin synthesis: PKS1 from *Colletotrichum orbiculare* releases T4HN, which is immediately converted by the T4HN reductase^57^ while PKS1 of the black yeast *Exophiala dermatitidis* releases the hexaketide AT4HN, which is modified to T4HN by YG1, an ortholog of AYG1.^58^ In a phylogenetic tree based on amino acid identity (Figure 2B), *C. antarcticus* PKS1 groups with PKS from other Dothideomycetes (68%–73%), and *E. dermatitidis* PKS1 (48%), *K. petricola* PKS1 (47%), and *B. cinerea* PKS13 (49%). For all these fungi, the requirement of the PKS for DHN melanin formation was experimentally shown.^21^, ^49^, ^59^, ^60^, ^61^, ^62^ The close relationship of CaPKS1 with these PKSs suggests that CaPKS1 is involved in DHN melanogenesis as well.

To see whether CaPKS1 is a functional protein, putatively producing the same product as the PKS1 from the black fungus *K. petricola*, both PKSs were heterologously expressed from plasmid DNA in the budding yeast *Saccharomyces cerevisiae*. For this, intron‐free sequences of the *C. antarcticus* and *K. petricola* genes were fused to a galactose‐inducible promoter in yeast expression vectors. Thus, gene expression in plasmid‐carrying *S. cerevisiae* strains occurred during cultivation in presence of galactose but not in presence of glucose (Figure 2C, Figure S2). As expected, the expression of either *capks1* or *kppks1* alone did not induce the production of any metabolite, as the *S. cerevisiae* PPT (LYS5) cannot activate PKSs.^63^ Consistent with this, the co‐expression of the PKSs with the Sfp‐type phosphopantetheinyl transferase PPT1 from *K. petricola* resulted in the production and accumulation of similar looking yellowish metabolites. As T4HN is colorless and its oxidation product flaviolin reddish‐brown, KpPKS1 and CaPKS1 may release another polyketide that must be converted in an additional step to T4HN as demonstrated in *A. fumigatus*,^54^ *E. dermatitidis*,^58^ and *B. cinerea* ^49^ (Figure S1). With the “yellowish‐green hydrolases” CaYGH1 and CaYGH2, *C. antarcticus* possesses two proteins orthologous to AYG1, EdYG1, and BcYGH1, which may produce T4HN from a longer polyketide. However, the PKSs may also release all YWA1, AT4HN, and T4HN in different quantities as recently found for *A. alternata* PksA.^47^ Although the two PKSs may release different products in *K. petricola* and *C. antarcticus*, CaPKS1 has been demonstrated to be a functional protein with the potential to provide the phenolic precursor for the formation of DHN melanin.

### 3.3 Development of protocols for protoplast‐mediated transformation of *C. antarcticus*

Gene functions in the native organism can be studied by targeted mutation or overexpression approaches as far as the organism is genetically amendable. Different strategies for the transformation of fungi exist.^64^ In all fungi, but especially in black fungi, the cell wall constitutes the main barrier for the uptake of recombinant DNA. Given that protoplasts can be obtained from the fungus of interest, their transformation allows for different options, as different types of DNA (linear, circular plasmid DNA) as well as ribonucleoproteins (RNPs) for CRISPR/Cas9‐mediated genome editing can be introduced.^65^

**FIGURE 3:**
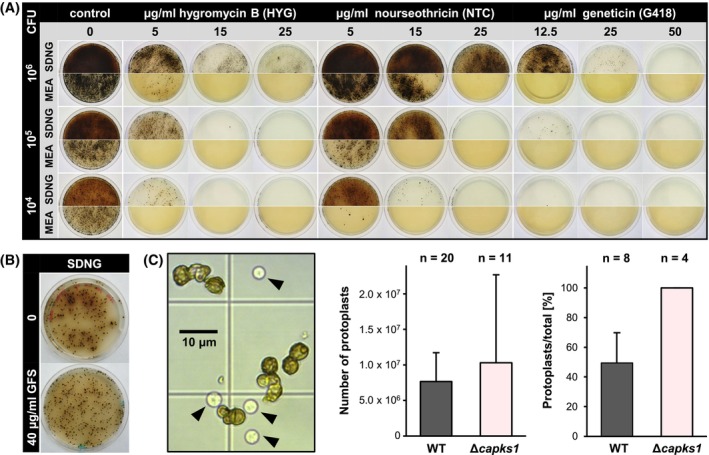
Prerequisites for genetic transformation of *C. antarcticus*. (A). *C. antarcticus* wild type is sensitive to the aminoglycosides hygromycin B, nourseothricin and geneticin. MEA and SDNG agar plates were inoculated with different numbers of colony‐forming units (CFU) and incubated for 3 months at 15°C in darkness. (B). Glufosinate ammonium (GFS) does not prevent growth of *C. antarcticus*. SDNG agar without or supplemented with GFS was inoculated with 2 × 10^3^ CFU and incubated for 3 months at 15°C in the dark. (C). Protoplasts from highly melanized *C. antarcticus* cells can be obtained. Incubation of biomass in osmotically stabilized buffer with cell wall‐degrading enzymes yielded protoplasts (black arrows) for the melanized WT after 14–16 h and for the non‐melanized Δ*capks1* mutant already after 3–4 h. Protoplast numbers and the protoplast cell ratio were calculated from *n* approaches. Mean values and standard deviations are shown.

Protocols for the generation and transformation of protoplasts of *K. petricola* have been optimized over the years^21^, ^31^, ^40^ and can serve as a blueprint for the transformation of other black fungi. First, it was verified that *C. antarcticus* did grow well on media, that is, SDNG (synthetic‐defined nitrate glucose) and MEA (malt extract agar), that are used for the transformation and transformant maintenance of *K. petricola* (Figure 3A,B). Further, protoplasts from the highly melanized *C. antarcticus* cells were obtained following the same protocol, that is, incubation of cells for 16 h at 27°C in an osmotically stabilized buffer containing a mixture of cell wall‐degrading enzymes. As for *K. petricola*, cells and protoplasts cannot completely be separated by size from each other, but protoplasts are easily identified by their clear cell wall and turgid shape in contrast to the heavily melanized cells (Figure 3C). The number of protoplasts obtained from melanized *C. antarcticus* biomass was 7.7 × 10^6^ on average. The protoplast/cell ratio ranged between 0.4 and 0.6.

Independent of the transformation method, it is necessary to select transformants, that is, cells that have been taken up and integrated the recombinant DNA into their genomes. Selection can be achieved using toxic compounds (selective agents) and using donor DNA carrying a cassette for conferring resistance to the corresponding toxic compound. Frequently used selection marker systems in fungi, including *K. petricola*, are hygromycin B (HYG)/hygromycin phosphotransferase (HPH), nourseothricin (NTC)/nourseothricin acetyltransferase (NAT1), geneticin (G418)/neomycin phosphotransferase (NPTII), or glufosinate ammonium (GFS)/phosphinothricin N‐acetyltransferase (BAR).^31^, ^64^ However, fungi may exhibit different or even no sensitivity to these compounds, and thus, appropriate selective agents and their optimal concentrations for inhibiting growth of the respective fungus must be figured out. Therefore, growth of *C. antarcticus* on MEA and SDNG supplemented with various concentrations of HYG, NTC, or G418 was evaluated by plating 10^4^, 10^5^, or 10^6^ cells per Petri dish. Overall, the *C. antarcticus* cell viability after incubation for 3 months decreased with increasing concentrations of HYG, NTC, and G418 (Figure 3A). Remarkably, the fungus showed higher sensitivity to the compounds on MEA compared with SDNG. As expected from observations made for *K. petricola*, the sensitivity correlated with the numbers of cells plated. Thus, concentrations of 25 μg/mL of the three selective agents prevented growth of 10^5^ cells/Petri dish on both media. However, it must be considered that protoplasts might be more sensitive to the selective agents than cells. Growth of *C. antarcticus* on SDNG after 6 weeks of incubation was unaffected by 40 μg/mL of GFS, a dose preventing growth of *K. petricola* in the amino acid‐free SDNG medium (Figure 3B). The observed ineffectiveness of GFS may be ascribed to either the tolerance of *C. antarcticus* to the tested concentration or the instability of the chemical compound over the long incubation time.

With the possibility to obtain protoplasts of *C. antarcticus* and the identification of appropriate compounds (HYG, NTC, G418) and selective concentrations, the prerequisites for the transformation of *C. antarcticus* with recombinant DNA were fulfilled.

### 3.4 Deletion of *capks1* by a CRISPR/Cas9‐assisted replacement approach

With supporting evidence that CaPKS1 provides precursors for the DHN melanin, a targeted replacement strategy for *capks1* was considered to generate a non‐melanized *C. antarcticus* strain.

**FIGURE 4:**
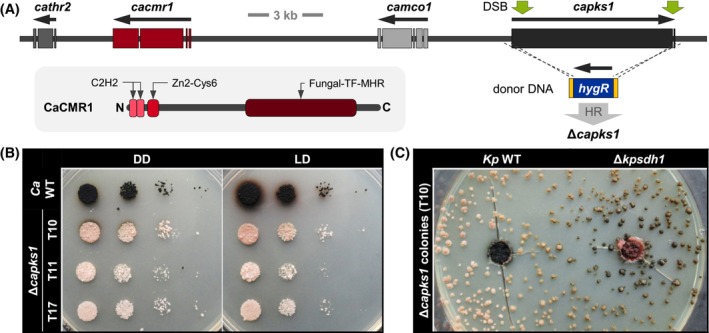
Deletion of *pks1* in *C. antarcticus* abolishes melanization. (A). The PKS1‐encoding gene is physically linked with other putative melanogenic genes. The protein domains (C2H2 zinc finger [PF00096], fungal Zn(2)‐Cys^6^ binuclear cluster domain [PF00172], fungal transcription factor regulatory middle homology region [cd12148]) of the putative transcription factor CaCMR1 are shown. The *capks1* coding region was deleted from the genome by a CRISPR/Cas9‐assisted replacement approach. Two double strand breaks (DSBs; green arrows) were introduced for triggering homologous recombination (HR) using the provided donor DNA (a hygromycin resistance cassette flanked by 75‐bp‐long sequences homologous to the *capks1* flanking sequences) as template. Three hygromycin Δ*capks1* mutants were obtained (see Figure S4 for details). (B). The three independent Δ*capks1* mutants exhibit an identical phenotype. Cell suspensions (10^4^, 10^3^, 10^2^, 10^1^ CFU per droplet) of the wild‐type strain (*Ca* WT) and the deletion mutants were dropped onto MEA and incubated for 5 weeks at 15°C in constant darkness (DD) or in 12 h light/12 h darkness (LD). (C). Pathway intermediates secreted by Δ*kpsdh1* restore melanization of the Δ*capks1* mutant. MEA was inoculated with ~500 CFU of Δ*capks1* and incubated for 3 months at 15°C in darkness. Then, two agar plugs were replaced by agar plugs covered with biomass of *K. petricola* A95 (*Kp* WT) and of a mutant that secretes DHN pathway intermediates (Δ*kpsdh1*), respectively. The picture was taken after additional incubation for 2 weeks at 15°C in darkness.

When exploring the genomic location of *capks1*, it was found that *capks1* is physically linked with further genes putatively associated with DHN melanogenesis (Figure 4A). Clustering of DHN melanogenic genes is frequently observed. Thereby, all genes (e.g., *A. fumigatus*), few genes (e.g., *B. cinerea*), or even none (e.g., *N. crassa*) are physically linked in the genome.^66^ *Cathr2*, *cacmr1*, and *capks1* are organized in the same manner as in *A. alternata* and other Dothideomycetes, which is in accordance with their close relationships. *Cathr2* and *camco1* might be involved in the synthesis of DHN and its polymerization, respectively. *Cacmr1* encodes a putative transcription factor, whose orthologs such as *C. orbiculare* CMR1,^67^ *A. alternata* CMR1,^68^ and *B. cinerea* SMR1^49^ regulate the expression of the DHN melanogenic genes. In accordance with the studied orthologs, CaCMR1 contains the two conserved DNA‐binding domains in its N‐terminal region, which is a C2H2‐zinc finger and a Zn(2)‐Cys^6^ binuclear cluster. Based on the sequence similarities, the activity of CaCMR1 may regulate the expression of all the other melanin‐encoding genes in *C. antarcticus*.

A gene deletion approach was designed to replace *capks1* with a resistance cassette by homologous recombination (HR) (Figures 4A and S4A). For triggering DNA repair in that specified genomic location, two double strand breaks (DSBs) at both ends of *capks1* should be introduced by the CRISPR/Cas9 technology. Consequently, the DSBs would be repaired by HR when a resistance cassette flanked by sequences homologous to the 5′‐ and 3′‐noncoding regions of *capks1* is provided as repair template (donor DNA). The plasmid pAMA/tRNA‐*capks1* ^PS1^‐*capks1* ^PS4^ for the transient expression of Cas9 and two *capks1*‐specific sgRNAs was cloned. It contained *cas9* fused to a nuclear localization signal with the codon usage of *A. niger* and under control of the regulatory sequences of *A. nidulans tef1*, and a tRNA‐sgRNA cassette under control of the regulatory sequences of *A. fumigatus* U3 for transcription by the RNA polymerase III. This plasmid was developed for genome editing in *Aspergillus* spp.^41^ but is also used in *K. petricola*.^31^ Donor DNAs, containing a hygR or a natR cassette flanked by 75‐bp‐long sequences homologous to the *C. antarcticus* genome, were generated using primers binding via the 3′ region to the resistance cassette in pNDR‐OGG plasmids, and having 75‐bp‐long 5′ overhangs for attaching the *C. antarcticus* sequences (Figure S4A). Protoplasts were transformed with the circular plasmid and linear donor DNA, either mediating hygR or natR, plated on osmotically stabilized medium (MEAS) and overlaid after 24 h with medium supplemented with the respective selective agents to obtain final concentrations of 25 μg/mL for HYG and 5 μg/mL for NTC, respectively. For control, protoplasts were transformed with H_2_O and overlaid with selective medium (control for background growth) or non‐selective medium (control for recovery of protoplasts). Initially, protoplasts could regenerate a cell wall when incubated in the osmotically stabilized media used for the transformation of *K. petricola*, and growth of recovered wild‐type cells was suppressed by the concentrations of the selective agents that were used for *K. petricola*. For the transformation samples, numerous dark and light‐colored colonies appeared after 4 months of incubation at 15°C on the top of the selective medium. For confirmation of the transformation events resulting in hygR or natR, mainly light‐colored but also some black colonies were transferred to MEA supplemented with HYG (25 μg/mL) and NTC (5 μg/mL), respectively. While most of the light‐colored cells grew with HYG, proofing them as hygR transformants, the ones from the transformation with the natR approach failed to grow on NTC‐containing medium. In a few cases, the streaking of light‐colored cells from the transformation plates onto HYG‐containing agar yielded a mixture of wild‐type‐like and non‐melanized colonies. For these transformants, cells from non‐melanized colonies were repeatedly spread and isolated to clear out melanized cells. The transformation of wild‐type protoplasts with the *capks1*‐hygR replacement fragment yielded non‐melanized colonies in four different experiments (Table S5), demonstrating the reproducibility of the approach.

In total, 27 hygR Δ*capks1* transformants from two transformation experiments were submitted to diagnostic PCR for detecting the replacement of *capks1* by the hygR cassette, as consequence of HR events at 5′ and 3′ of the DSBs/*capks1*. For this, primers binding in the hygR cassette were combined with those binding up and downstream of *capks1* in the genome. Further, *capks1* was detected with primers binding in the native coding regions in between the two Cas9 cutting sites. The expected amplicon pattern was observed in the three transformants T10, 11, and 17 (Figure S4C), resulting in a deletion rate of 11% (3 out of 27 tested). Remarkably, all strains tested were not melanized indicating the absence of a functional allele of *capks1*. However, in 24 strains, either the HR events could not be detected and/or *capks1* was detected (data not shown) suggesting that the Cas9‐mediated DSBs were repaired by non‐homologous end‐joining rather than by HR resulting in random mutations. A drop assay was performed to compare the growth phenotype of the three verified Δ*capks1* mutants with that of the wild type on MEA when incubated in complete darkness (DD) or in a diurnal light–dark (LD) cycle (Figure 4B). All three mutants exhibited a light pinkish pigmentation and moreover growth rates comparable with those of the wild type. Therefore, three independent hygR Δ*capsk1* mutants were generated that are not melanized. This demonstrates that genetic engineering of *C. antarcticus* is feasible (Figure S3), though it is very lengthy (8 to 9 months) due to the very slow growth rates.

### 3.5 Restoration of melanization in Δ*capks1* by DHN precursors secreted by *K. petricola*

The genetic complementation of the Δ*capks1* mutants by adding back the *capks1* was envisaged as control. To prevent the disruption of other genes by an ectopic integration of a *capks1* expression construct, a targeted knock‐in strategy was considered. As no suitable noncoding/intergenic regions for the targeted integration were available, a strategy was designed to reinsert *capks1* in its native genomic region under control of its native 5′‐noncoding region for comparable transcription levels in the complemented strains and the wild type. A construct was cloned containing the coding region of *capks1* with 0.364 kb of the 5′‐noncoding region upstream of the *B. cinerea gluc* terminator and a natR cassette (Figure S5A). Upon introduction of a DSB in the resistance gene (*hph*) of the Δ*capks1* locus, the sequences homologous to the 5′‐noncoding sequence of *capks1* (0.364 kb) and the *trpC* promoter present in both hygR and natR cassettes (0.357 kb) would allow for HR events, replacing the former hygR cassette by the *capks1*‐natR expression construct. Protoplasts of the Δ*capks1* mutant were obtained and transformed with the circular pAMA/tRNA‐*hph* ^PS1^ for expression of Cas9 and a *hph*‐specific sgRNA, and the linear *capks1*‐natR construct isolated by digestion from pN‐*capks1* ^COMIL^. However, no natR transformants were obtained. To rule out mistakes in the complementation strategy, the same strategy was used to complement a natR *K. petricola* Δ*pks1* mutant (Figure S5B). Numerous colonies with a wild‐type‐like pigmentation grew on the transformation medium. Four of them were isolated and analyzed by diagnostic PCR, which confirmed the envisaged HR events in three strains. These strains produced DHN melanin like the wild type and grew with HYG but not with NTC as expected (Figure S5B). Thus, the pursued complementation strategy was correct, indicating that the failure in obtaining complemented Δ*capks1* strains was rather due to the hypersensitivity of the non‐melanized *C. antarcticus* cells and protoplasts to NTC (similar observations were made for the natR Δ*capks1* transformants) or to the transformation procedure itself.

Nonetheless, the ability to restore melanogenesis in Δ*capks1* was assessed through delivery of DHN precursors. As can be seen in the drop assay, the edges of the pinkish Δ*capks1* colonies next to the *C. antarcticus* wild‐type colonies turned dark (Figure 4B). In another assay, the Δ*capks1* mutant was co‐cultivated with the *K. petricola* wild‐type and the Δ*kpsdh1* mutant, which secretes pathway intermediates due to the mutation of the scytalone dehydratase‐encoding gene (Figure 4C). The colonies around the Δ*kpsdh1* mutant but not those surrounding the wild type became pigmented, suggesting that those Δ*capks1* colonies took up the brownish metabolites secreted by Δ*kpsdh1* and converted them into DHN melanin. By both approaches, it was shown that the other DHN melanogenic genes in Δ*capks1* mutants remained intact, and the *C. antarcticus* wild type unlike *K. petricola* secretes DHN pathway intermediates into the medium. These metabolites either remain colorless or are taken up again during incubation in constant darkness. Under diurnal cycles (LD), they are oxidized to their unusable shunt products, resulting in brownish halos around melanized colonies. The fact that the secretion of the same dark pigments in both DD and LD was prevented in Δ*capks1* demonstrates that they all derive from CaPKS1 as the key enzyme in DHN melanogenesis.

### 3.6 Replacement of the carotenogenic genes by a H2B‐GFP expression construct

**FIGURE 5:**
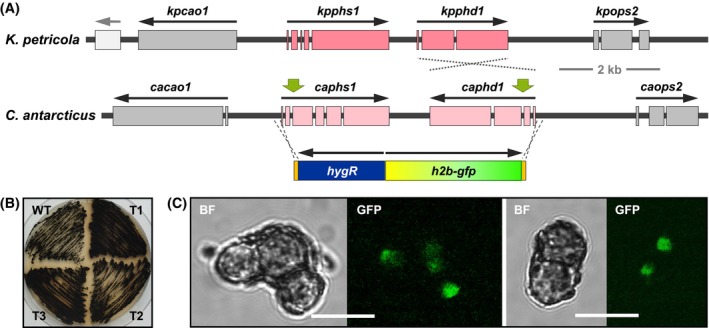
Insertion of an expression construct in the carotenogenic gene cluster of *C. antarcticus*. (A). The putative carotenogenic genes *caphs1* and *caphd1*. The four physically linked genes in the genomes of *K. petricola* (GenBank: MT859420.1) and *C. antarcticus* (Cryan3 scaffold 55: 150,000‐163,000) are shown: *phs1*—phytoene synthase/lycopene cyclase; *phd1*—phytoene desaturase; *cao1*—carotenoid oxygenase; *ops2*—microbial opsin. For the expression of a histone 2B‐GFP fusion protein in *C. antarcticus*, adjacent *caphs1* and *caphd1* were replaced by a hygR cassette‐containing expression construct. Cas9 cutting sites (green arrows) and the donor DNA with 75‐bp‐long sequences homologous to the 5′‐noncoding regions of both genes (orange bars) are shown. See Figure S6 for further details. (B). Deletion of *caphs1* and *caphd1* does not alter the pigmentation. Wild‐type and three Δ*caphs1*‐*caphd1* mutants with the *h2b‐gfp* construct (CA‐0021) were cultivated for 3 weeks on MEA at 25°C in DD. (C). Δ*caphs1*‐*caphd1* mutants with the *h2b‐gfp* expression construct exhibit green fluorescence in the nuclei. GFP fluorescence in cells of transformant T1 (CA‐0021) is shown. BF—brightfield, scale bars – 10 μm.

The deletion of *capks1* causing the inability to produce precursors for DHN melanin resulted in mutants with a light pinkish pigmentation. Similar observations, that is, that other pigments become visible after blocking melanization, have been made in other fungi. Most striking is the intense pink to reddish pigmentation of non‐melanized *K. petricola* mutants (Δ*kppks1*) (Figure S5B). Other black fungi lacking the ability to produce DHN melanin, including *E. dermatitidis*, exhibit a light pinkish pigmentation comparable to that of Δ*capks1* mutants. While the pigmentation of *E. dermatitidis* Δ*pks1* mutants was found to be more pronounced during cultivation in the light than in the dark,^69^ the light conditions did not significantly affect the pigmentation of the Δ*capks1* mutant (Figure 4B) or the Δ*kppks1* mutant (data not shown). The pink to reddish pigmentation in the latter mutant is due to the accumulation of carotenoids, as the deletion of the carotenogenic genes abolishes their synthesis resulting in albino mutants.^21^ Carotenogenic genes are contained in the genome of many DHN melanin‐producing fungi^70^ and are often clustered with genes encoding a carotenoid oxygenase for the formation of retinal and a green light‐driven proton pump, such as in *Fusarium fujikuroi* and *B. cinerea*.^71^, ^72^ To see whether *C. antarcticus* has the capability to produce carotenoids, BlastP searches using the sequences of the *K. petricola* carotenogenic enzymes were performed. This led to the identification of a bifunctional phytoene synthase/lycopene cyclase (CaPHS1, 49% aa identity with KpPHS1) and a phytoene desaturase (CaPHD1, 59% aa identity with KpPHD1) (Figure 5A, Table S1). While *phs1* and *phd1* have the same orientation in most fungi including *K. petricola*, *caphs1* and *caphd1* share a terminator region.

To validate carotenoid production in *C. antarcticus*, an approach was considered to delete both adjacent carotenogenic genes in a single step. pAMA/tRNA‐*caphs1* ^PS1^‐*caphd1* ^PS1^ was cloned for the expression of ribonucleoproteins introducing DSBs in the 5′‐coding regions of the two genes. Two types of donor DNA were used to replace the genes either by a resistance cassette only (hygR or natR) or by a resistance cassette fused to a cassette for the expression of a histone 2B‐green fluorescent protein (H2B‐GFP) fusion protein. Each donor DNA contained 75‐bp‐long sequences homologous to the 5′‐noncoding regions of *caphs1* and *caphd1* (Figures 5A and S6). The transformation of *C. antarcticus* wild‐type protoplasts with the hygR‐containing donor DNA yielded 17 transformants for the hygR cassette only (Δ*caphs1‐caphd1*; CA‐0017) and five hygR transformants for the *h2b‐gfp* expression construct (*h2b‐gfp*; CA‐0021). To detect the desired replacement events in the resistant transformants, diagnostic PCR analyses were carried out as specified in Figure S6. This revealed the correct HR events and the absence of *caphs1‐caphd1* in 13 out of 17 (CA‐0017) and five out of five transformants (CA‐0021). These deletion rates of 76% and 100% were significantly higher than that for the deletion of *capks1* (deletion rate of 11%), for which only non‐melanized transformants were analyzed by diagnostic PCR. Thus, these deletion approaches proved the effectiveness of editing the *C. antarcticus* genome using HYG/*hph* as selection system. Unfortunately, no transformation of wild‐type protoplasts with the Δ*caphs1‐caphd1*‐natR fragment was carried out in parallel. This might have shown if the failure to obtain non‐melanized natR transformants was due to an increased sensitivity to NTC.

The obtained Δ*caphs1‐caphd1* mutants displayed a wild‐type‐like phenotype but putatively lack carotenoids (Figure 5B). The H2B‐GFP fusion protein, expected to be localized in the nuclei of *C. antarcticus*, could be detected by confocal fluorescence microscopy (Figure 5C). The signal intensity was rather low—likely as consequences of the thick melanized cell wall and slow metabolism—but the approach showed that the *B. cinerea*‐optimized *gfp* under control of the *A. nidulans* P*oliC* is expressed in *C. antarcticus* and thus is suitable for studying its cell biology. Furthermore, this experiment revealed that *C. antarcticus* cells contain a single nucleus. The attempt to stain the nuclei with the DAPI dye failed due to unspecific binding of the dye to the cell wall, suggesting that the dye cannot enter the cell to bind the DNA.

However, the transformation of Δ*capks1* protoplasts with pAMA/tRNA‐*caphs1* ^PS1^‐*caphd1* ^PS1^ and a *capks1*‐natR replacement fragment did not yield any transformants. Therefore, the question of whether the light pinkish pigmentation of the Δ*capks1* mutants is due to accumulation of carotenoids remains unanswered.

### 3.7 Growth of *C. antarcticus* strains is attenuated by visible light

**FIGURE 6:**
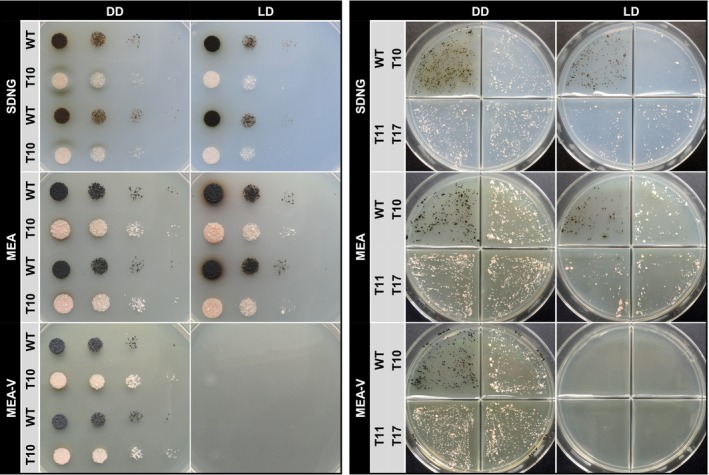
Melanized and non‐melanized *C. antarcticus* strains are equally sensitive to visible light. The wild‐type strain and Δ*capks1* mutants were cultured for 6 weeks at 15°C in DD or LD conditions. Solidified media on the left were inoculated with droplets of cells suspensions (10^4^, 10^3^, 10^2^, 10^1^ CFU per droplet) (see the same plates on white background in Figure S7 for brownish metabolites secreted by the wild type). The media on the right were inoculated with ~500 CFU per Petri dish compartment. MEAV in contrast to MEA does not contain glucose and peptone.

Initial results on the *C. antarcticus* wild‐type and non‐melanized Δ*capks1* mutants indicated that their phenotypes differ when incubated in DD (complete darkness) in comparison with LD (diurnal light–dark) conditions (Figure 4B). To confirm this observation and to see whether it is related with the culture medium used, serial dilutions of wild‐type and the Δ*capks1* cells were dropped onto three different solid media. MEA, used previously, is a rich medium containing glucose and peptone as additional carbon and nitrogen sources, allowing fast growth. SDNG is a synthetic medium, containing vitamins and trace elements, and glucose and nitrate as carbon and nitrogen source, respectively. MEAV is an alternative malt extract medium which is used for the cultivation of Antarctic fungi^73^; it lacks additional carbon and nitrogen sources (Figures 6 and S7). After 6 weeks of incubation in DD, the wild‐type and the Δ*capks1* mutant exhibited similar growth rates across all media. But during exposure to LD cycles, both strains showed decreased cell viability on MEA and SDNG, and neither strain did grow on the glucose‐ and peptone‐free MEAV. The secretion of brownish pigments by the wild‐type colonies was affected by light on MEA as observed before, but also by the medium. Thus, the colonies secreted on SDNG agar more metabolites in DD compared with LD. Brownish metabolites, likely intermediate products of DHN melanin, were observed being taken up by the mutant strains in DD. Conversely, a rapid oxidation of the secreted metabolites by light prevented their uptake by mutants under LD conditions. Assuming that in this experiment cells in droplets may have shielded underlying cells from light, single‐cell growth assays were conducted with the wild‐type and the three Δ*capks1* strains. For this, cells were spread onto agar in compartmentalized Petri dishes to prevent metabolites from wild‐type colonies from impacting on the non‐melanized mutants (Figure 6). Consistent observations of reduced (MEA/SDNG) or absent growth (MEAV) of both wild‐type and mutant strains under LD conditions confirmed light sensitivity across all strains. These observations suggest that light may act as a stress‐inducing factor for *C. antarcticus*, yet growth can be sustained when more nutrients are readily available.

Sensitivity to visible light has not been reported for *C. antarcticus* so far. The fungus lives in association with cryptoendolithic microbial communities in Antarctic sandstone rocks.^1^ These communities typically display a vertical zonation pattern, assumed to be formed in response to the surrounding sunlight regime. As sandstones are sedimentary rocks with quartz grains, incident sunlight can penetrate inside the rock and be transmitted to deeper layers due to mineral transparency, interacting with both the photosynthetic and non‐photosynthetic components of microbial communities.^74^ Black fungi are typically found in the first colonized zone, located just a few millimeters below the rock crust. This area has been estimated to receive up to 10% of the surface light flux,^75^ and therefore, dark melanins in these fungi are believed to act as light filters, alleviating photo‐oxidative damage caused by the excessive solar irradiation on the rest of the community.^76^ Observations made from our experiments imply that the sensitivity of *C. antarcticus* to visible light is an inherent trait and not contingent upon DHN melanin production. However, the meaning of this effect in the natural habitat of the fungus remains elusive and requires further investigation.

### 3.8 The deletion of *capks1* affects the cell wall organization

Melanins are best known for their function to protect cells from harmful UV radiation, but they may have further specific functions in fungi depending on their habitats and life cycles. Thus, DHN melanin‐containing cell walls may function as mechanical barrier and give strength and shape to infection or reproduction structures, thereby protecting the fungal cells from abiotic and biotic stress factors and contributing to dispersal and survival of the reproductive units.^77^

**FIGURE 7:**
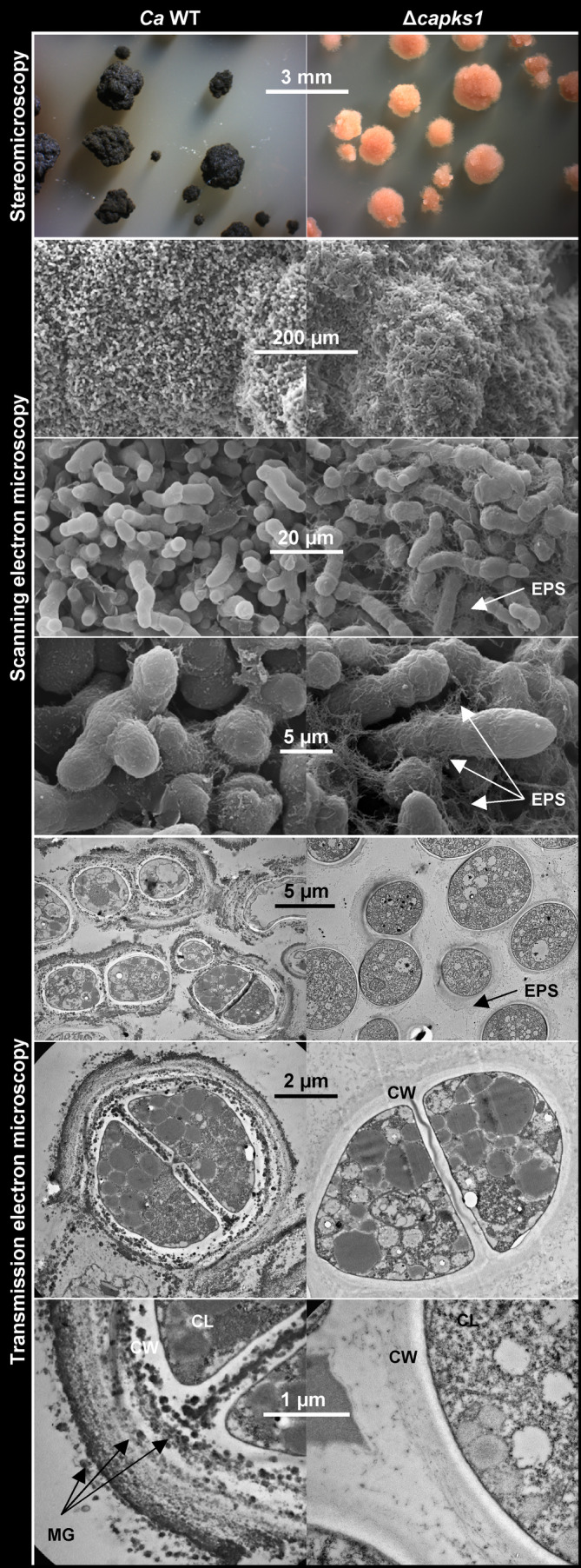
Deletion of *pks1* in *C. antarcticus* alters the organization of the cell wall. Microscopic analyses of melanized wild‐type and non‐melanized Δ*capks1* colonies from biofilm cultures (MEA). EPS—extracellular polysaccharides; CW—cell wall; CL—cell lumen; MG—melanin granules.

To see whether DHN melanin or its absence alters the morphology of *C. antarcticus* cells and colonies, optical and electron microscopy were applied (Figure 7). Stereomicroscopic analyses revealed a consistent colony‐forming ability in the Δ*capks1* mutant, with colony sizes comparable with those of the wild type. The distinctive trait of Δ*capks1* colonies was the light pinkish pigmentation and a fluffier appearance. Assuming that these pinkish pigments are carotenoids, they may be incorporated in the membranes of *C. antarcticus* as found in *K. petricola*. Carotenoid production is supposed to occur in both mutant and wild type yet being hidden in the latter due to the dark cell wall. Scanning electron microscopy demonstrated that the cell aggregation in Δ*capks1* colonies resembles that of the wild‐type colonies. Both melanized and non‐melanized cells are covered with extracellular polysaccharides (EPS), though the Δ*capks1* mutant produce more EPS as was found for the non‐melanized *K. petricola* Δ*pks1* mutant.^33^ The EPS has been characterized before in several melanized strains of *C. antarcticus*,^1^ but not in the one used in this study. Therefore, composition and amounts of EPS might be compared between wild‐type and the non‐melanized Δ*capks1* mutant to clarify whether the absence of DHN melanin affects the EPS production in *C. antarcticus* as well. Transmission electron microscopy revealed wild‐type and Δ*capks1* cells with intact plasma membranes and cell walls. Consistent with previous studies on the wild‐type strain,^78^, ^79^ the deposition of DHN melanin manifested as tiny, highly electron‐dense granules (approx. 20–100 nm in diameter) in the outer layers of the cell wall and on top. Conversely, no electron‐dense depositions were observed in the cell walls of the non‐melanized mutant. These structural changes in the cell wall are thus associated with DHN melanin. Interestingly, the arrangement of DHN melanin in the cell walls of *C. antarcticus* and *K. petricola* differs. Dark melanin granules are found throughout the multi‐layered cell wall of *C. antarcticus*, while a smoother DHN layer is observed in the outer wall of budding *K. petricola* cells.^30^, ^33^

In summary, melanin‐deficient and carotenoid‐deficient *C. antarcticus* mutants were successfully generated by a genetic engineering approach in this study. Utilization of these mutants in further phenotypic studies will gain new insights into the role of DHN melanin in the extraordinary survival capabilities of *C. antarcticus*.

## 4 CONCLUSIONS AND PERSPECTIVES

The Antarctic black fungus *C. antarcticus* is a slow growing fungus originating from a cold desert—the closest terrestrial analogue to conditions on early Mars. Its ability to resist absolute (*par excellence*) extreme environments was repeatedly demonstrated. The role of the dark pigment melanin for survivability of this fungus has been postulated. Here, we further improved *C. antarcticus* as a test organism by implementing genetic engineering tools for the targeted mutation of genes in its genome. The tools developed and optimized in the model black fungus *K. petricola* were successfully transferred to the *C. antarcticus* system demonstrating their reliability and efficiency. Only minimal technical modifications in the procedure were necessary. But the largest difference in genetically manipulating the two black fungi is the time needed to complete this task: 3 to 4 weeks in *K. petricola* and 8 to 9 months in *C. antarcticus*. Using the annotated genome sequence of *C. antarcticus* available at the JGI, genes putatively involved in the synthesis of DHN melanin and carotenoids were identified allowing to propose the synthesis pathways. To generate mutants defective in DHN melanogenesis, *capks1* encoding the key enzyme of the pathway was deleted. Resulting deletion mutants were non‐melanized and exhibited a light pinkish pigmentation, perhaps due to the accumulation of carotenoids. Thus, the carotenogenic genes were targeted by a deletion approach in the wild‐type and the non‐melanized mutants. While the deletion of the two adjacent genes in the wild type (Δ*caphs1‐phd1*) was successful, no Δ*capks1/*Δ*caphs1‐phd1* mutants were obtained. Therefore, the link between the pinkish pigments and the carotenogenic genes could not be established in this study, but both DHN melanin‐ and carotenoid‐deficient mutants have become available for functional and chemical characterization.

Due to its polyextremotolerant nature, *C. antarcticus* has become a prominent test organism in the field of astrobiology. With the now introduced availability of CRISPR/Cas9‐mediated genome editing, *C. antarcticus* becomes an even better test organism for astrobiology and related research.

## Supporting information


**Data S1.** Supporting information.
