# Supplementary material for: Deletion of the polyketide synthase‐encoding gene pks1 prevents melanization in the extremophilic fungus Cryomyces antarcticus
Source: IUBMB Life. 2024 Jul 16;76(12):1072–90. doi: 10.1002/iub.2895 (PMC11580375; doi:10.1002/iub.2895)
Supplement: Supplementary file 1 — Data S1. Supporting information. [file IUB-76-1072-s001.pdf]

# Deletion of the polyketide synthase-encoding gene *pks1* prevents melanization in the extremophilic fungus *Cryomyces antarcticus*

**Ilaria Catanzaro [1,2], Ruben Gerrits [1], Ines Feldmann [1], Anna A. Gorbushina [1,3], Silvano Onofri [2], and Julia Schumacher [1,3] ***

*1 Bundesanstalt für Materialforschung und -prüfung (BAM), Berlin, Germany*

*2 Università degli Studi della Tuscia, Viterbo, Italy*

*3 Freie Universität Berlin, Germany*

* Corresponding author: Julia.Schumacher@bam.de

SUPPLEMENTARY FIGURES .....2

Figure S1. The proposed synthesis pathway of DHN melanin in *C. antarcticus*. .....2

Figure S2. Heterologous expression of *C. antarcticus* and *K. petricola* genes in *S. cerevisiae*. .....3

Figure S3. The generation of *C. antarcticus* deletion mutants takes eight to nine months. .....4

Figure S4. Generation and verification of *C. antarcticus pks1* deletion mutants. .....5

Figure S5. Complementation of deletion mutants by reintroducing the gene into the native locus. .....6

Figure S6. Replacement of the carotenogenic genes in *C. antarcticus*. .....7

Figure S7. The *C. antarcticus* wild type but not the Δ*capks1* mutant secretes brownish pigments. .....8

SUPPLEMENTARY TABLES .....9

Table S1. *Cryomyces antarcticus* melanogenic and carotenogenic genes. .....9

Table S2. Accession numbers of proteins associated with DHN melanogenesis from other fungi. .....10

Table S3. Oligonucleotides used in this study. .....11

Table S4. Plasmids cloned in this study. .....14

Table S5. Transformations of *C. antarcticus* and *K. petricola* carried out in this study. .....15

SUPPLEMENTARY REFERENCES .....16

# SUPPLEMENTARY FIGURES

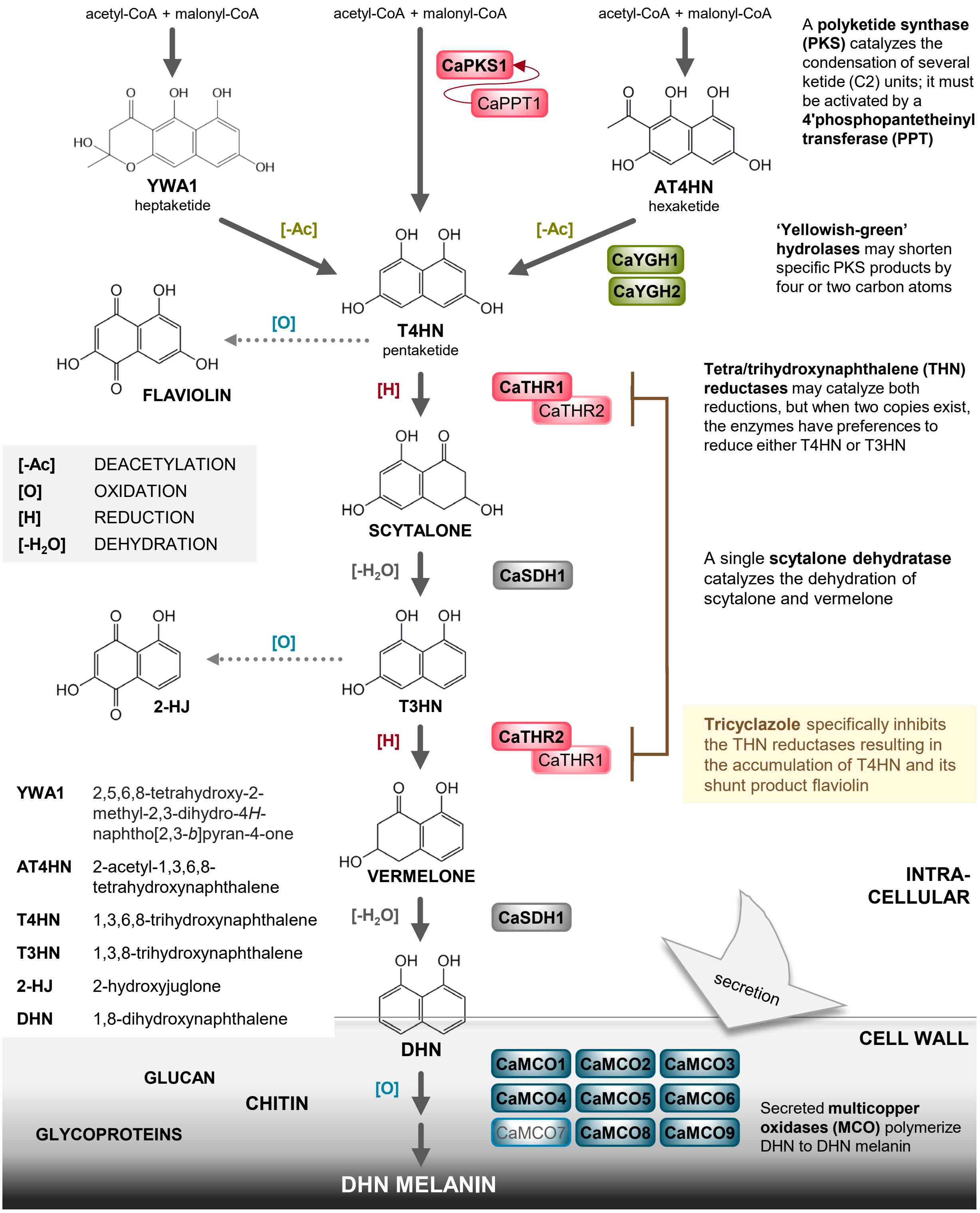


**Figure S1. The proposed synthesis pathway of DHN melanin in *C. antarcticus*.**

The putative DHN melanogenic genes of *C. antarcticus* were identified in the genome database based to their similarity with the ones described in *A. fumigatus* (Figure 1A). It is supposed that the PKS of *C. antarcticus* releases a longer polyketide (yellowish pigment) that is in turn deacetylated by CaYGH1/2 to T4HN. Nine MCO/laccases were identified due to their typical domain structure, for eight MCOs a N-terminal signal peptide for secretion was predicted (bold letters) (Figure 1B).

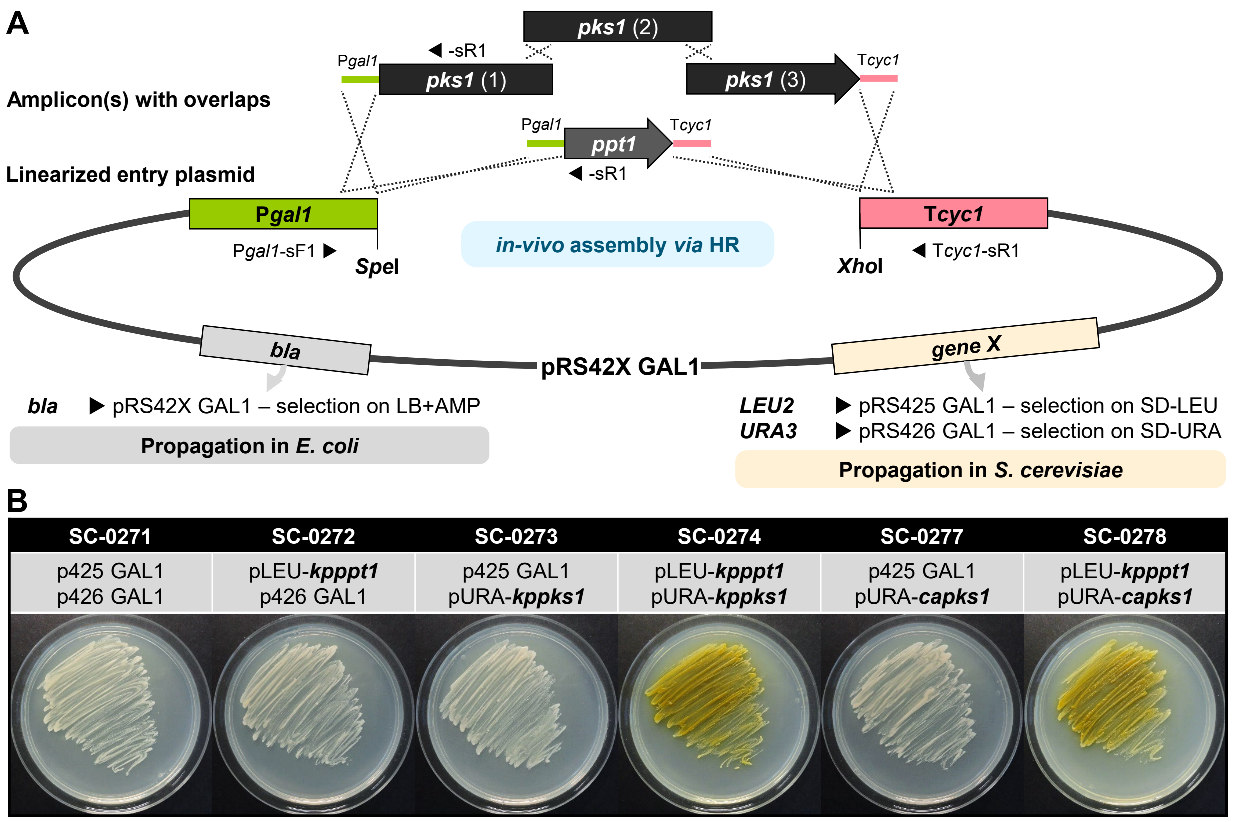


**Figure S2. Heterologous expression of *C. antarcticus* and *K. petricola* genes in *S. cerevisiae*.**

**A. Cloning of the expression vectors.** The genes of interest (*goi*) with their stop codons were amplified from genomic DNA as one fragment (*kpppt1*; no intron) or three fragments (*kppks1*, *capks1*; each gene with one intron) using primers for generating 25-bp-long overlaps with P*gal1* and T*cyc1*. Assembly in a *Spe*I+*Xho*I-digested entry plasmid of the pRS42X GAL1 series (Mumberg et al., 1994) was carried out in the LEU and URA-auxotrophic *S. cerevisiae* strain FY834 (Table S4). Plasmid DNA from LEU-prototrophic (p425 GAL1 derivatives, selected on SD-LEU) or URA-prototrophic (p426 GAL1 derivatives, selected on SD-URA) colonies was extracted and introduced into *E. coli*. Ampicillin-resistant *E. coli* colonies were screened by PCR for insert-carrying plasmids by combining a primer binding in a regulatory sequence (P*gal1*-sF1) with a primer binding in the insert (*goi*-sR1). The three cloned expression vectors, containing intron-free *kpppt1*, *kppks1* or *capks1*, were sequenced with primers P*gal1*-sF1 and T*cyc1*-sR1 for verification of the sequence. **B. Formation of the yellowish PKS products requires PPT activity (KpPPT1).** *S. cerevisiae* strain FY834 was co-transformed with plasmids containing *LEU2* or *URA3* and selected for LEU and URA prototrophy by using glucose-containing SD-LEU-URA (SD/GLU-LU) medium. Empty plasmids (p425 GAL1 and p426 GAL1) were transformed for mediating prototrophy of the control strains. Prototrophic *S. cerevisiae* colonies containing different combinations of *K. petricola* and *C. antarcticus* genes were streaked onto galactose-containing SD-LEU-URA (SD/GAL-LU) agar for induction of the galactose-responsive promoter (P*gal1*). Pictures were taken after three days of incubation at 30 °C in the dark. See also Figure 2C.

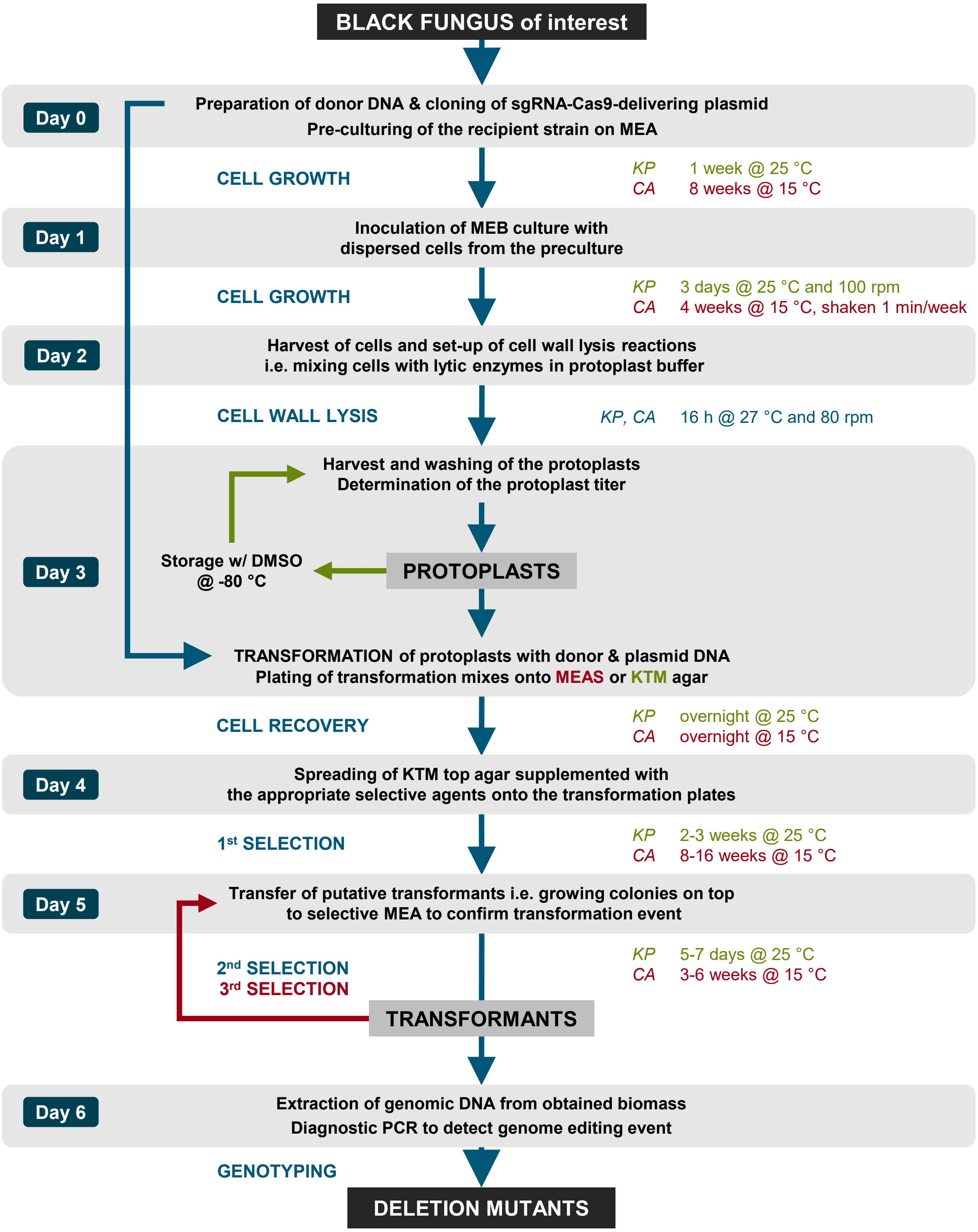


**Figure S3. The generation of *C. antarcticus* deletion mutants takes eight to nine months.**

Work and time flow of the generation and transformation of protoplasts, and the isolation and genotyping of resistant transformants of *K. petricola* [*KP*, in green] (Noack-Schönmann et al., 2014; Voigt et al., 2020; Erdmann et al., 2022) and *C. antarcticus* [*CA*, in red] (this study). The option to preserve *C. antarcticus* protoplasts with DMSO at -80°C has not been evaluated. Examples are shown: protoplasts and cells (Figure 3C), putative transformants on transformation plates and resistance transformants growing on selective medium (Figure S4B), and deletion mutants confirmed by diagnostic PCR to lack the gene of interest (Figure S4C).

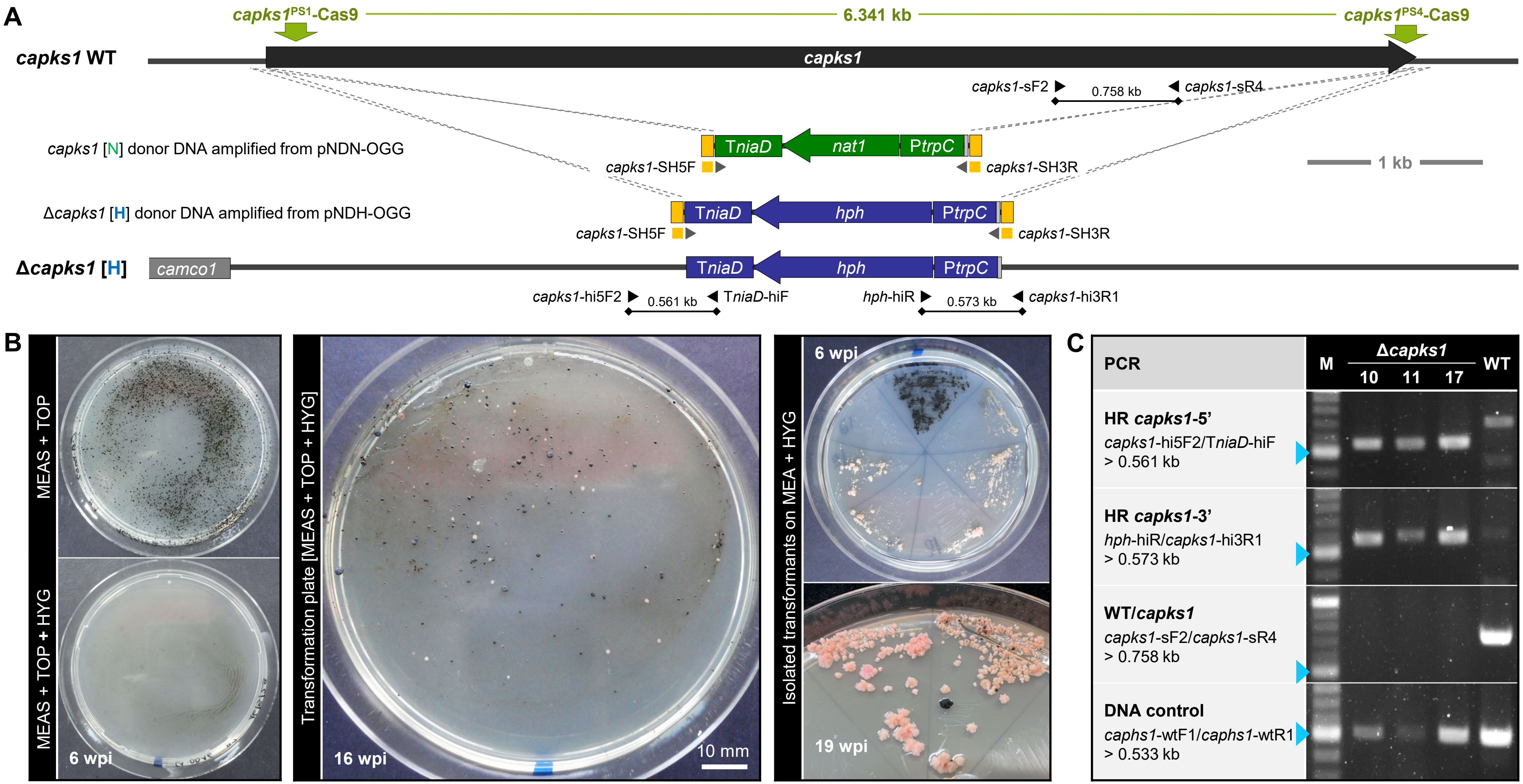


**Figure S4. Generation and verification of *C. antarcticus pks1* deletion mutants.**

**A. Strategy for replacing *capks1* with a resistance cassette.** Wild type protoplasts were transformed with the circular sgRNA/Cas9-delivering plasmid pAMA/tRNA-*capks1*$^{PS1}$-*capks1*$^{PS4}$ and linear donor DNA that was amplified with the primer pair *capks1*-SH5F/*capks1*-SH3R from pNDN-OGG or pNDH-OGG. The primers attached 75-bp-long sequences homologous to the 5'- and 3'-noncoding regions of *capks1* to the resistance cassettes (orange bars). The Cas9 cutting sites are indicated as green arrows. **B. The transformation with donor DNA containing a hygromycin resistance (hygR) cassette yielded hygR transformants with different pigmentation.** Left: for control, protoplasts were transformed without DNA showing that protoplasts regenerates when no hygromycin (25 µg/ml HYG) was added. Middle: transformation plate with black and pinkish colonies. Right: examples of isolated transformants growing on HYG-containing medium. **C. Diagnostic PCR revealed three non-melanized Δ*capks1* mutants.** Homologous recombination (HR) events at 5' and 3' of *capks1* and the absence of *capks1* were detected by using the primer pairs shown. Blue triangles refer to the 0.5-kb-large band of the used DNA ladder (M).

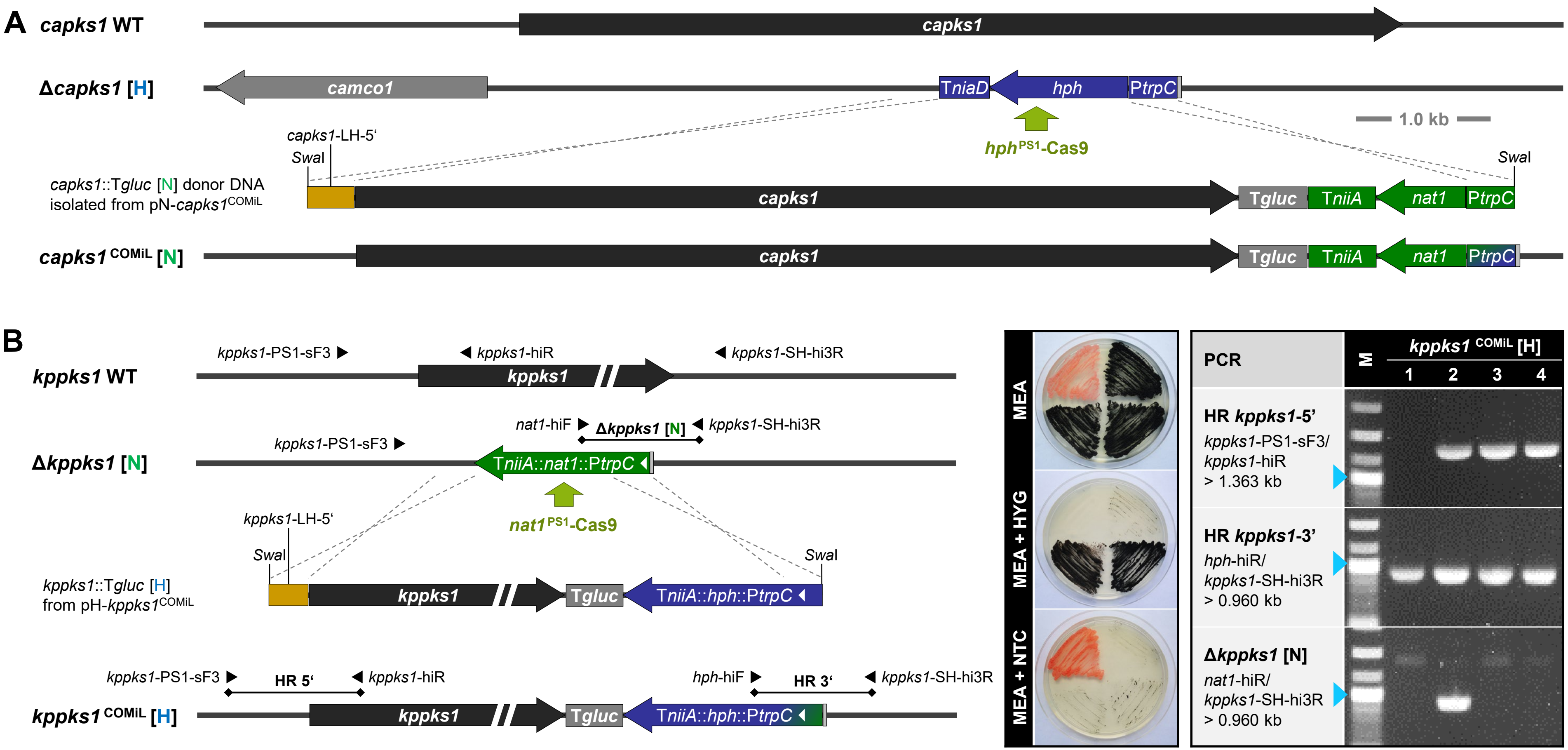


**Figure S5. Complementation of deletion mutants by reintroducing the gene into the native locus.**

**A. Strategy pursued for replacing the hygromycin resistance (hygR) cassette in the Δ*capks1* mutant by a *capks1* expression construct.** Protoplasts of Δ*capks1* were transformed with the circular sgRNA/Cas9-delivering plasmid pAMA/tRNA-*hph*$^{\mathrm{PS1}}$ and the linear expression construct *capks1*::T*gluc* [N] isolated from pN-*capks1* by digestion with *Swa*I. It was envisaged that the DSB in the hygR cassette would be repaired by homologous recombination via the 5’-noncoding region of *capks1* and P*trpC*, which is present in both resistance cassettes. However, nourseothricin-resistant (natR) transformants could not be isolated. **B. Validation of the strategy in *K. petricola* by complementing the natR Δ*kppks1* mutant.** Left: Δ*kppks1* protoplasts were transformed with pAMA/tRNA-*nat1*$^{\mathrm{PS1}}$ for inserting a DSB in *nat1* and the linear *kppks1*::T*gluc* [H] construct as donor DNA. Middle: Obtained hygR transformants [growth on MEA with 25 µg/ml of hygromycin (HYG)] had a black pigmentation and lost the ability to grow on MEA containing 5 µg/ml of nourseothricin (NTC). Wild type A95 (top right), melanin-deficient Δ*kppks1* (top left) and two transformants (T3, T4; bottom) were cultivated for five days. Right: The correct replacement of the natR cassette by the *kppks1* expression construct in transformants *kppks1*$^{\mathrm{COMiL}}$ T3 and T4 was verified by diagnostic PCR (transformants 1 to 4 were tested). Light blue triangles highlight the 1-kb-band of the used DNA ladder (M).

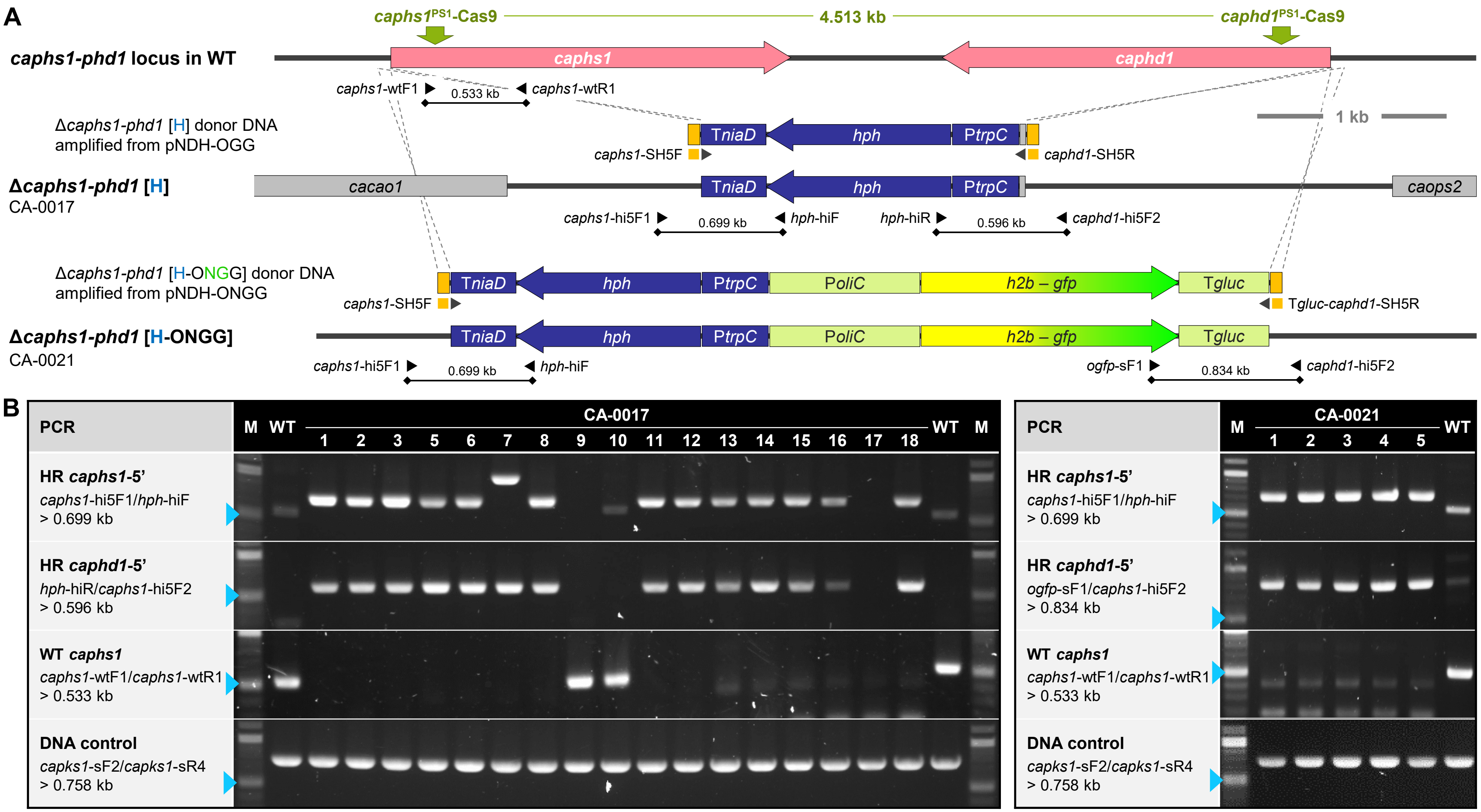


**Figure S6. Replacement of the carotenogenic genes in *C. antarcticus*.**

**A. Strategies for replacing adjacent *caphs1* and *caphd1* in detail.** Wild type protoplasts were transformed with the circular pAMA/tRNA-*caphs1*$^{PS1}$-*caphd1*$^{PS1}$ and linear donor DNA, which was amplified from pNDH-OGG and pNDH-ONGG with the primer pairs *caphs1*-SH5F/*caphd1*-SH5R and *caphs1*-SH5F/ T*gluc*-*caphd1*-SH5R, respectively. Homologous recombination via the 75-bp-long homologous sequences (orange bars) results in the replacement of both genes. **B. Detection of replacement in obtained hygR transformants.** DNA from the hygR transformants was submitted to diagnostic PCR using the primer pairs as indicated. Light blue triangles highlight the 0.5-kb-band of the DNA ladder (M).

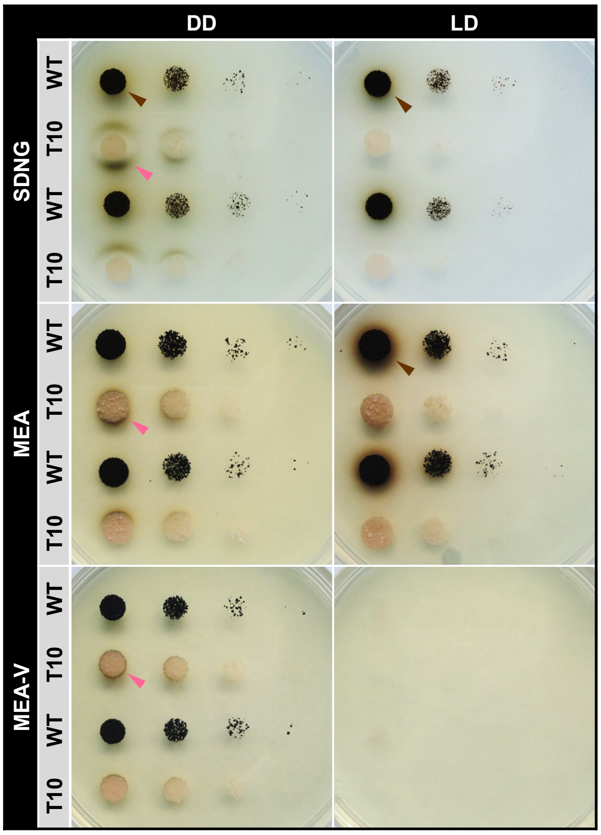


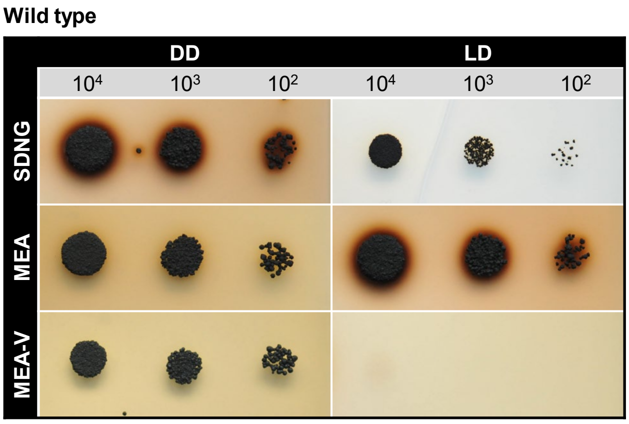


**Figure S7. The *C. antarcticus* wild type but not the Δ*capks1* mutant secretes brownish pigments.**

On the left: Pictures of the *C. antarcticus* cultures shown in Figure 6 were taken on a white background to demonstrate the secretion of brownish pigments by the wild type, and the uptake and conversion of these pigments by the Δ*capks1* mutant when cultivated under DD conditions in contrast to LD conditions. Brown arrows: secretion of brownish water-soluble metabolites by WT; pink arrows: conversion of the brownish metabolites to black DHN melanin by non-melanized mutant. On the right: Pictures of a drop assay in which cell suspensions of the wild type (alone) were spotted on the same media and incubated for three months in the two different light conditions. Note the staining of the media (SDNG in DD and MEA in LD) due to the secretion of the brownish metabolites in the absence of Δ*capks1* colonies.

# SUPPLEMENTARY TABLES

**Table S1. *Cryomyces antarcticus* melanogenic and carotenogenic genes.**

| Name | Description | JGI protein ID | Size | Note | Location | Possible allelic (secondary) variant |
|---|---|---|---|---|---|---|
| **CaPPT1** | Phosphopantetheinyl transferase | **Cryan3\|30097** | 337 aa | primary allele | Scaffold_01: 1742250-1743437 | n/a |
| **CaPKS1** | Polyketide synthase | **Cryan3\|585847** | 2165 aa | primary allele | Scaffold_06: 122131-129243 | Protein ID **456827** on scaffold_42 |
| **CaYGH1** | Yellowish-green hydrolase | **Cryan3\|833091** | 419 aa | primary allele | Scaffold_01: 630851-632345 | Protein ID **858952** on scaffold_40 |
| **CaYGH2** | Yellowish-green hydrolase | **Cryan3\|27039** | 397 aa | primary allele | Scaffold_01: 1579623-1581597 | Protein ID **548851** on scaffold_52 |
| **CaTHR1** | T4HN reductase | **Cryan3\|836228** | 270 aa | primary allele | Scaffold_03: 205816-206693 | Protein ID **995051** on scaffold_28 |
| **CaTHR2** | T3HN reductase | **Cryan3\|586390** | 266 aa | primary allele | Scaffold_06: 147010-148334 | Protein ID **1031432** on scaffold_42 |
| **CaSDH1** | Scytalone dehydratase | **Cryan3\|954298** | 195 aa | primary allele | Scaffold_33: 504911-505643 | Protein ID **543413** on scaffold_51 |
| **CaCMR1** | Transcription factor | **Cryan3\|586214** | 964 aa | primary allele | Scaffold_06: 141665-145152 | Protein ID **1031431** on scaffold_42 |
| **CaFET1** | Multicopper oxidase/ferroxidase | **Cryan3\|837159** | 601 aa | primary allele | Scaffold_04: 520366-522519 | Protein ID **710496** on scaffold_9 |
| **CaMCO1** | Multicopper oxidase/laccase | **Cryan3\|586223** | 595 aa | primary allele | Scaffold_06: 132368-134973 | Protein ID **1068762** on scaffold_42 |
| **CaMCO2** | Multicopper oxidase/laccase | **Cryan3\|839253** | 544 aa | primary allele | Scaffold_06: 927319-929072 | Protein ID **957745** on scaffold_67 |
| **CaMCO3** | Multicopper oxidase/laccase | **Cryan3\|856845** | 644 aa | primary allele | Scaffold_35: 413320-415153 | Protein ID **861533** on scaffold_48 |
| **CaMCO4** | Multicopper oxidase/laccase | **Cryan3\|860958** | 619 aa | primary allele | Scaffold_46: 313375-315394 | Protein ID **1070979** on scaffold_60 |
| **CaMCO5** | Multicopper oxidase/laccase | **Cryan3\|1057704** | 563 aa | primary allele | Scaffold_02: 1111648-1113613 | Protein ID **956007** on scaffold_45 |
| **CaMCO6** | Multicopper oxidase/laccase | **Cryan3\|452356** | 619 aa | primary allele | Scaffold_41: 247514-250025 | Protein ID **1115966** on scaffold_45<br>Protein ID **1034174** on scaffold_148 |
| **CaMCO7** | Multicopper oxidase/laccase | **Cryan3\|453744** | 553 aa | primary allele | Scaffold_41: 320863-323412 | Protein ID **1115935** on scaffold_45 |
| **CaMCO8** | Multicopper oxidase/laccase | **Cryan3\|846371** | 802 aa | primary allele | Scaffold_15: 894719-898717 | n/a |
| **CaMCO9** | Multicopper oxidase/laccase | **Cryan3\|50117** | 1007 aa | primary allele | Scaffold_10: 1004053-1008308 | n/a |
| **CaPHS1** | Phytoene synthase/lycopene cyclase | **Cryan3\|1119942** | 628 aa | primary allele | Scaffold_55: 156344-158724 | Protein ID **1104509** on scaffold_26 |
| **CaPHD1** | Phytoene desaturase | **Cryan3\|1070518** | 635 aa | primary allele | Scaffold_55: 153713-155792 | Protein ID **273264** on scaffold_26 |
| **CaCAO1** | Carotenoid oxygenase | **Cryan3\|1011935** | 738 aa | primary allele | Scaffold_55: 159475-162523 | Protein ID **993463** on scaffold_26 |
| **CaOPS2** | Green light-absorbing microbial opsin | **Cryan3\|563816** | 322 aa | primary allele | Scaffold_55: 150397-151843 | Protein ID **1065685** on scaffold_26 |

Genes and proteins from *Cryomyces antarcticus* strain CBS 116301 (DOE Joint Genome Institute) are shown. Many of the smaller scaffolds are very similar to larger scaffolds and are predicted to constitute an alternate or secondary haplotype. Thus, 'primary alleles' and 'secondary alleles' gene model tracks are given in the genome portal (https://mycocosm.jgi.doe.gov/mycocosm/home). The primary alleles are listed only for providing a non-redundant set of genes putatively involved in DHN melanin formation.

**Table S2. Accession numbers of proteins associated with DHN melanogenesis from other fungi.**

| | ***Aspergillus fumigatus*** **Af293** [Eurotiomycetes] | | ***Alternaria alternata*** **ATCC 66981** [Dothideomycetes] | | ***Neurospora crassa*** **OR74A** [Sordariomycetes] | | ***Botrytis cinerea*** **B05.10** [Leotiomycetes] | |
|---|---|---|---|---|---|---|---|---|
| | Name [size] | Locus tag GenBank acc. | Name [size] | Protein ID JGI | Name [size] | Locus tag GenBank acc. | Name [size] | Locus tag GenBank acc. |
| **Phosphopantetheinyl transferase (PPT)** | NpgA [359 aa] | Afu2g08590 **XP_755193.1** | PPT1 [375 aa] | **Alalte1\|106450** | PPT-1 [348 aa] | NCU00581 **XP_965721.2** | PPT1 [354 aa] | BCIN_06g04100 **ATZ50945.1** |
| **Polyketide synthase (PKS)** | Alb1 [2146 aa] | Afu2g17600 **XP_756095.1** | PKSA [2161 aa] | **Alalte1\|111952** | PER-1 [2206 aa] | NCU03584 **XP_960586.3** | PKS12 [2147 aa] | BCIN_02g08770 **ATZ47616.1** |
| **Polyketide synthase (PKS)** | *no hit* | *no hit* | *no hit* | *no hit* | *no hit* | *no hit* | PKS13 [2138 aa] | BCIN_03g08050 **ATZ48614.1** |
| **Yellowish-green hydrolase (YGH)** | Ayg1 [406 aa] | Afu2g17550 **XP_756090.1** | AYGA [420 aa] | **Alalte1\|115293** | PKH-2 [430 aa] | NCU05821 **XP_960081.1** | YGH1 [410 aa] | BCIN_02g04360 **ATZ47119.1** |
| **Yellowish-green hydrolase (YGH)** | *no hit* | *no hit* | AYGB [403 aa] | **Alalte1\|105009** | PKH-1 [487 aa] | NCU01903 **XP_965534.3** | *no hit* | *no hit* |
| **Tetrahydroxynaphthalene reductase (T4HNR)** | Arp2 [273 aa] | Afu2g17560 **XP_756091.1** | BRM3 [268 aa] | **Alalte1\|112254** | PKR-2 [268 aa] | NCU06905 **XP_959252.1** | BNR2 [266 aa] | BCIN_03g08100 **ATZ48619.1** |
| **Trihydroxynaphthalene reductase (T3HNR)** | *no hit* | *no hit* | BRM2 [267 aa] | **Alalte1\|111954** | PKR-1 [282 aa] | NCU09390 **XP_011395293.1** | BNR1 [289 aa] | BCIN_04g04800 **ATZ49315.1** |
| **Scytalone dehydratase (SDH)** | Arp1 [168 aa] | Afu2g17580 **XP_756093.1** | BRM1 [185 aa] | **Alalte1\|105968** | SCY-1 [174 aa] | NCU07823 **XP_962944.1** | SCD1 [167 aa] | BCIN_03g08110 **ATZ48620.1** |
| **C2H2-Zn(2)Cys(6) transcription factor** | RegA [866 aa] | Afu1g17640 **XP_753130.1** | CMR1 [1010 aa] | **Alalte1\|111953** | SAH-12 [1241 aa] | NCU02787 **XP_963943.3** | SMR1 [938 aa] | BCIN_02g08760 **ATZ47614.1** |
| **Multicopper oxidase/laccase (MCO)** | Abr1 [664 aa] | Afu2g17540 **XP_756089.2** | n/a | n/a | n/a | n/a | n/a | n/a |
| **Multicopper oxidase/laccase (MCO)** | Abr2 [587 aa] | Afu2g17530 **XP_756088.2** | n/a | n/a | n/a | n/a | n/a | n/a |
| **Multicopper oxidase/ferroxidase (FET)** | FetC [592 aa] | Afu5g03790 **XP_747965.2** | FET1 [586 aa] | **Alalte1\|114711** | LCC3 [693 aa] | NCU03498 **XP_955835.1** | FET1 [615 aa] | BCIN_02g02780 **ATZ46936.1** |
| **Phytoene synthase/lycopene cyclase (PHS1)** | *no hit* | *no hit* | [583 aa] | **Alalte1\|108710** | AL-2 [602 aa] | NCU00585 **XP_965725.3** | PHS1 [610 aa] | BCIN_01g04560 **ATZ45729.1** |
| **Phytoene desaturase (PHD1)** | *no hit* | *no hit* | [628 aa] | **Alalte1\|108713** | AL-1 [595 aa] | NCU00552 **XP_964713.1** | PHD1 [602 aa] | BCIN_01g04550 **ATZ45728.1** |
| **Carotenoid oxygenase (CAO1)** | *no hit* | *no hit* | [707 aa] | **Alalte1\|108711** | *no hit* | *no hit* | CAO1 [649 aa] | BCIN_01g04570 **ATZ45730.1** |
| **Green light-absorbing microbial opsin (OPS2)** | *no hit* | *no hit* | [308 aa] | **Alalte1\|108712** | [292 aa] | NCU01735 **XP_011393497.1** | BOP2 [338 aa] | BCIN_01g04540 **ATZ45726.1** |

**Table S3. Oligonucleotides used in this study.**

| Name | Sequence (5'→ 3') | Features (5'→ 3')/binding sites |
|---|---|---|
| ***Kpppt1*-*Pgal1*-F** | aggagaaaaaaccccggattctaga-ATGGCCTACACCACCGGCGATG | *S. cerevisiae* P*gal1* – *kpppt1* |
| ***Kpppt1*-*Tcyc1*-R** | taagcgtgacataactaattacatg-TCAGTCGAGACAACAACATTTGCC | *S. cerevisiae* T*cyc1* – *kpppt1* |
| ***Kpppt1*-wtR** | GAGCGAAGTGTGCATGGTGTGTG | *K. petricola ppt1* coding region |
| ***Kppks1*-*Pgal1*-F** | aggagaaaaaaccccggattctaga-ATGGAGGAAGTCTACGTGTTCGG | *S. cerevisiae* P*gal1* – *kppks1* start of coding region |
| ***Kppks1*-*Tcyc1*-R** | taagcgtgacataactaattacatg-TTAGCCCTGAATGGCTTCACGAATG | *S. cerevisiae* T*cyc1* – *kppks1* end coding region |
| ***Kppks1*-Y1R** | AGCATTTTCAGAGCGTTCATCAACATATATAC | *K. petricola pks1* coding region |
| ***Kppks1*-Y2F** | ggctgtatatatgttgatgaacgctctgaaaatgctc-CAATGAAATGACAAAACTGAGAAATGCAAAG | *K. petricola pks1* coding region deleted for intron |
| ***Kppks1*-wtF2** | GCCGATCTGGCATACACCACCAC | *K. petricola pks1* coding region |
| ***Kppks1*-wtR2** | GTCCGAGACGCCGTTGATGCATG | *K. petricola pks1* coding region |
| **P*gal1*-sF1** | GTCGCGTTCCTGAAACGCAGATG | *S. cerevisiae* P*gal1* in p42X GAL1 |
| **T*cyc1*-sR1** | GGACCTAGACTTCAGGTTGTCTAAC | *S. cerevisiae* T*cyc1* in p42X GAL1 |
| ***Capks1*-*Pgal1*-F** | aggagaaaaaaccccggattctaga-ATGTCCAACATCGTACTTTTCGGC | *S. cerevisiae* P*gal1* – *capks1* start of coding region |
| ***Capks1*-*Tcyc1*-R** | aagcgtgacataactaattacatg-ctatgcggagagaccgagtccctgtctgat gagacggccaagggtttcacc-GTGTTCATCACGCATCATCGTGAAG | *S. cerevisiae* T*cyc1* – *capks1* end of coding region with deleted intron |
| ***Capks1*-sF1** | GGCTGAGGAGACACTCCACTTGG | *C. antarcticus pks1* coding region |
| ***Capks1*-sF2** | GAACACTGAGGCCACTGCTTCCG | *C. antarcticus pks1* coding region |
| ***Capks1*-sF3** | GCCGCTATCCAACTCGCTTGTAC | *C. antarcticus pks1* coding region |
| ***Capks1*-sF4** | GCGAGTATGCTGCTCTTCACGTTG | *C. antarcticus pks1* coding region |
| ***Capks1*-sF5** | GTTGCTTGGTCACCATTGCCCAG | *C. antarcticus pks1* coding region |
| ***Capks1*-sR5** | GTTCCAATACGGTGCAACTTCGTCG | *C. antarcticus pks1* coding region |
| ***Capks1*-sR1** | GTGATTCTTGCGAAGAGCCTCCG | *C. antarcticus pks1* coding region |
| ***Capks1*-sR2** | GTGTTACCACCGGCAGCAGAGAAG | *C. antarcticus pks1* coding region |
| ***Capks1*-sR3** | GTCGGAAGCGTTGACGATGAAGCC | *C. antarcticus pks1* coding region |
| ***Capks1*-sR4** | GAGCGGCACCATCGTATTGTGAG | *C. antarcticus pks1* coding region |
| **pFC334-F1** | GGTCATAGCTGTTTCCGCTGA | pFC332/pFC902 and derivatives |
| **pFC334-R1** | TGATTCTGCTGTCTCGGCTG | pFC332/pFC902 and derivatives |
| **Pa*fU3*-sF1** | GCTTGAGGTTAGCGCACTCGCTAG | *A. fumigatus* P*U3* in pFC902 and derivatives |

| Name | Sequence (5'→ 3') | Features (5'→ 3')/binding sites |
|---|---|---|
| **P*tef1*-sR1** | CGTTCGAGAGCATGATCAGCAC | *A. nidulans* P*tef1* in pFC332 and derivatives |
| ***Capks1*-tRNA-PS1F** | gctaccgcgctctcagcgtg-GTTTTAGAGCTAGAAATAGCAAGTTAAAAT | *C. antarcticus pks1*-PS1 – sgRNA in pFC902 |
| ***Capks1*-tRNA-PS1R** | cacgctgagagcgcggtagc-TGCATCATCCGTGAATCGAAC | *C. antarcticus pks1*-PS1 – tRNA in pFC902 |
| ***Capks1*-tRNA-PS4F** | gccagtgctaacattgtcac-GTTTTAGAGCTAGAAATAGCAAGTTAAAAT | *C. antarcticus pks1*-PS4 – sgRNA in pFC902 |
| ***Capks1*-tRNA-PS4R** | gtgacaatgttagcactggc-TGCATCATCCGTGAATCGAAC | *C. antarcticus pks1*-PS4 – tRNA in pFC902 |
| ***Capks1*-SH5F** | cactcgctgcatttacgagatctatcgacctataccatatcatctacgaacgatccatcccctaaaataatcaa t-GCTAAGCGAGCGGGAGCTATCG | *C. antarcticus pks1*-5' (75 nt) – *B. cinerea* T*niaD* |
| ***Capks1*-SH3R** | agggtaccactccgcagcgtcaccaaggcactgtggaaaaagaagaatcacacatgtatctgtcgagaaggaag t-GAATCGGGAATGCGGCTCCACAG | *C. antarcticus pks1*-3' (75 nt) – *A. nidulans* P*oliC* |
| ***Capks1*-hi5F1** | GATCATCAGTCCACTTCCAGCCCG | *C. antarcticus pks1*-5' non-coding region |
| ***Capks1*-hi5F2** | GGCAGCATAGGAGGCACTGGATAC | *C. antarcticus pks1*-5' non-coding region |
| ***Capks1*-hi3R1** | GCTCCAACCAGATTGCCAATATCG | *C. antarcticus pks1*-3' non-coding region |
| ***Capks1*-hi3R2** | GCCACAGCTGTGATCGATGTAATC | *C. antarcticus pks1*-3' non-coding region |
| ***Caphs1*-tRNA-PS1R** | cggggtagtagagacaaggg-TGCATCATCCGTGAATCGAAC | *C. antarcticus phs1*-PS1 – tRNA in pFC902 |
| ***Caphs1*-tRNA-PS1F** | cccttgtctctactaccccg-GTTTTAGAGCTAGAAATAGCAAGTTAAAAT | *C. antarcticus phs1*-PS1 – gRNA in pFC902 |
| ***Caphd1*-tRNA-PS1R** | caggtgtatcgctaacattc-TGCATCATCCGTGAATCGAAC | *C. antarcticus phd1*-PS1 – tRNA in pFC902 |
| ***Caphd1*-tRNA-PS1F** | gaatgttagcgatacacctg-GTTTTAGAGCTAGAAATAGCAAGTTAAAAT | *C. antarcticus phd1*-PS1 – gRNA in pFC902 |
| ***Caphs1*-SH5F** | gctgtccacctccggaagcgctccatcccccgcttctcactctcatggcccggtaacgactacttttgacgcgc a-GCTAAGCGAGCGGGAGCTATCG | *C. antarcticus phs1*-5' (75 nt) – *B. cinerea* T*niaD* |
| ***Caphd1*-SH5R** | gctcccggcgcacgcagtcggacatcttcacatcaaaccgccgacatgtgattccctcaactcacgtataatgc g-GAATCGGGAATGCGGCTCCACAG | *C. antarcticus phd1*-5' (75 nt) – *A. nidulans* P*oliC* |
| ***Caphs1*-wtF1** | GCTCTGCTGCACTCACTGACCG | *C. antarcticus phs1* coding region |
| ***Caphs1*-wtR1** | GATTGGCAAGAGTGTGTTCGACAGTG | *C. antarcticus phs1* coding region |
| ***Caphs1*-hi5F1** | CTCGACGAGAGTCGTGGCTGCACTG | *C. antarcticus phs1*-5' non-coding region |
| ***Caphs1*-hi5F2** | GTCTGCACTGCTTCTCGCCAACACG | *C. antarcticus phs1*-5' non-coding region |
| ***Caphd1*-hi5F1** | GCCTTCAACCCGTTGTCCTAATGG | *C. antarcticus phd1*-5' non-coding region |
| ***Caphd1*-hi5F2** | GAGTGTACGCCTTTGCCATGCTGAG | *C. antarcticus phd1*-5' non-coding region |
| **T*niaD*-hiF** | GGTGCCAGATGTATCAGTGAGTCTG | *B. cinerea* T*niaD* |
| ***Hph*-hiF** | GTCTGGACCGATGGCTGTGTAGAAG | *hph* [hygromycin resistance (hygR, H) cassette] |
| ***Hph*-hiR** | GACAGACGTCGCGGTGAGTTCAG | *hph* [hygromycin resistance (hygR, H) cassette] |

| Name | Sequence (5'→ 3') | Features (5'→ 3')/binding sites |
|---|---|---|
| ***Nat1*-hiF** | CGGCGAGCAGGCGCTCTACATGAGC | *nat1* [nourseothricin resistance (natR, N) cassette] |
| ***Nat1*-hiR** | GTACCGGTAAGCCGTGTCGTCGAG | *nat1* [nourseothricin resistance (natR, N) cassette] |
| ***Ogfp*-sF1** | GGTGATGGTCCAGTCTTGCTC | *B. cinerea* optimized *gfp* coding region |
| ***Hph*-tRNA-PS1R** | cgtattgggaatccccgaac-TGCATCATCCGTGAATCGAAC | *hph*-PS1 – tRNA in pFC902 |
| ***Hph*-tRNA-PS1F** | gttcggggattcccaatacg-GTTTTAGAGCTAGAAATAGCAAGTTAAAAT | *hph*-PS1 – gRNA scaffold in pFC902 |
| ***Nat1*-tRNA-PS1R** | tggtcaggggcgggtccacc-TGCATCATCCGTGAATCGAAC | *nat1*-PS1 – tRNA in pFC902 |
| ***Nat1*-tRNA-PS1F** | ggtggacccgcccctgacca-GTTTTAGAGCTAGAAATAGCAAGTTAAAAT | *nat1*-PS1 – gRNA scaffold in pFC902 |
| **P*oliC*-pRS426-5F** | gccagggttttcccagtcacga-ccgcgg-atttaaat-TGCAGCTGTGGAGCCGCATTCCCG | pRS426-5F – *Sac*II – *Swa*I – *A. nidulans* P*oliC* |
| **T*gluc*-R1** | GATCTTGTTGGGGGGAAGGGGTTGTCAAATC | *B. cinerea* T*gluc* |
| **P*trpC*-SS-pRS426-3R** | ggataacaatttcacacaggaaaca-atttaaat-actagt-GATATTGAAGGAGCATTTTTTGGGC | pRS426-3R – *Swa*I – *Spe*I – *A. nidulans* P*trpC* |
| **T*niiA*-*Pac*I-T*gluc*-F** | caaccccttccccccaacaagat-taattaa-CAGATGCTGCTGGCAAGGTTACATC | *B. cinerea* T*gluc* – *Pac*I – *B. cinerea* T*niiA* |
| **T*gluc*-hiF** | CATACGTACATCTGATTTGACAACC | *B. cinerea* T*gluc* |
| **T*gluc*-sR2** | CCGCCCTCTTTTGTCTTCCGC | *B. cinerea* T*gluc* |
| **P*oliC*-sF2** | GGGAGACGTATTTAGGTGCTAGGG | *A. nidulans* P*oliC* |
| **pRS426-s5F2** | GTAGCGGTCACGCTGCGCGTAACC | upstream of cloning site in pRS42X derivatives |
| **pRS426-s3R2** | CATTAATGCAGCTGGCACGACAGG | downstream of cloning site in pRS42X derivatives |
| ***Capks1*-pRS426-5F** | gtaacgccagggttttcccagtcacg-atttaaat-GGCAGCATAGGAGGCACTGGATAC | pRS426-5F – *Swa*I – *capks1*-5' |
| ***Capks1*-T*gluc*-R** | taatcatacatcttatctacatacg-CTATGCGGAGAGACCGAGTCCCTG | *B. cinerea* T*gluc* – *capks1* |
| ***Kppks1*-pRS426-5F** | gtaacgccagggttttcccagtcacg-atttaaat-CAGGTCGGTTTCCGAATCTCAATG | pRS426-5F – *Swa*I – *kppks1*-5' |
| ***Kppks1*-COM-5R** | ACACCAGATACAGCCTCACCGTGACCAATGTTGGACTTGA | *K. petricola pks1* coding region |
| ***Kppks1*-T*gluc*-R** | taatcatacatcttatctacatacg-TTAGCCCTGAATGGCTTCACGAATG | *B. cinerea* T*gluc* – *kppks1* |
| ***Kppks1*-PS1-sF3** | GCTGGTAGAGTACGCTATATCCGC | *K. petricola pks1*-5' non-coding region |
| ***Kppks1*-wtF3** | GGCCGACTGAACTACTTCTTCAAG | *K. petricola pks1* coding region |
| ***Kppks1*-hiR** | GCTCCAGCTCTGAAAGCAATGCG | *K. petricola pks1* coding region |
| ***Kppks1*-SH-hi3R** | GAGTTAGATTCGAGACACTCCACCAG | *K. petricola pks1*-3' non-coding region |

Lowercase letters – 5' overhangs for cloning or for homologous recombination; uppercase letters – 3' part of the DNA oligonucleotides annealing to template DNA.

**Table S4. Plasmids cloned in this study.**

| Name [ID] (size) | Entry plasmid | Amplicon(s) | | | Assembly |
|---|---|---|---|---|---|
| **pLEU-*kpppt1*<br>[pEC0242]** (8.572 kb) | **p425 GAL1**<br>(Mumberg et al., 1994)<br>digested w/ *Spe*I + *Xho*I | ***kpppt1*** (1.101 kb)<br>primers: *kpppt1*-P*gal1*-F/*kpppt1*-T*cyc1*-R<br>template: *K. petricola* gDNA | | | *in vivo* |
| **pURA-*kppks1*<br>[pEC0243]** (12.897 kb) | **p426 GAL1**<br>(Mumberg et al., 1994)<br>digested w/ *Spe*I + *Xho*I | ***kppks1*-Y1** (0.339 kb)<br>primers: *kppks1*-P*gal1*-F/*kppks1*-Y2F<br>template: *K. petricola* gDNA | ***kppks1*-Y2** (2.893 kb)<br>primers: *kppks1*-Y1R/*kppks1*-wtR2<br>template: *K. petricola* gDNA | ***kppks1*-Y3** (4.039 kb)<br>primers: *kppks1*-wtF2/*kppks1*-T*cyc1*-R<br>template: *K. petricola* gDNA | *in vivo* |
| **pURA-*capks1*<br>[pEC0390]** (12.846 kb) | **p426 GAL1**<br>(Mumberg et al., 1994)<br>digested w/ *Spe*I + *Xho*I | ***capks1*-1** (2.418 kb)<br>primers: *capks1*-P*gal1*-F/*capks1*-sR2<br>template: *C. antarcticus* gDNA | ***capks1*-2** (2.461 kb)<br>primers: *capks1*-sF1/*capks1*-sR3<br>template: *C. antarcticus* gDNA | ***capks1*-3** (2.068 kb)<br>primers: *capks1*-sF2/*capks1*-T*cyc1*-R<br>template: *C. antarcticus* gDNA | *in vivo* |
| **pAMA/tRNA-<br>*capks1*$^{PS1}$-*capks1*$^{PS4}$<br>[pEC0256]** (16.670 kb) | **pFC332**<br>(Nødvig et al., 2015)<br>digested with *Pac*I | ***capks1*$^{PS1}$ tRNA** (0.589 kb)<br>primers: pFC334-F1/<br>*capks1*-tRNA-PS1R<br>template: pFC902 (Nødvig et al., 2018) | ***capks1*$^{PS1}$/*capks1*$^{PS4}$ tRNA** (0.191 kb)<br>primers: *capks1*-tRNA-PS1F/<br>*capks1*-tRNA-PS4R<br>template: pFC902 (Nødvig et al., 2018) | ***capks1*$^{PS4}$ tRNA** (0.427 kb)<br>primers: *capks1*-tRNA-PS4F/<br>pFC334-R1<br>template: pFC902 (Nødvig et al., 2018) | *in vitro* |
| **pAMA/tRNA-<br>*caphs1*$^{PS1}$-*caphd1*$^{PS1}$<br>[pEC0448]** (16.670 kb) | **pFC332**<br>(Nødvig et al., 2015)<br>digested with *Pac*I | ***caphs1*$^{PS1}$ tRNA** (0.589 kb)<br>primers: pFC334-F1/<br>*caphs1*-tRNA-PS1R<br>template: pFC902 (Nødvig et al., 2018) | ***caphs1*$^{PS1}$/*caphd1*$^{PS1}$ tRNA** (0.191 kb)<br>primers: *caphs1*-tRNA-PS1F/<br>*caphd1*-tRNA-PS1R<br>template: pFC902 (Nødvig et al., 2018) | ***caphd1*$^{PS1}$ tRNA** (0.427 kb)<br>primers: *caphd1*-tRNA-PS1F/<br>pFC334-R1<br>template: pFC902 (Nødvig et al., 2018) | *in vitro* |
| **pAMA/tRNA-*hph*$^{PS1}$<br>[pEC0461]** (16.499 kb) | **pFC332**<br>(Nødvig et al., 2015)<br>digested with *Pac*I | ***hph*$^{PS1}$ tRNA-A** (0.589 kb)<br>primers: pFC334-F1/*hph*-tRNA-PS1R<br>template: pFC902 (Nødvig et al., 2018) | ***hph*$^{PS1}$ tRNA-B** (0.427 kb)<br>primers: *hph*-tRNA-PS1F/pFC334-R1<br>template: pFC902 (Nødvig et al., 2018) | | *in vitro* |
| **pAMA/tRNA-*nat1*$^{PS1}$<br>[pEC0462]** (16.499 kb) | **pFC332**<br>(Nødvig et al., 2015)<br>digested with *Pac*I | ***nat1*$^{PS1}$ tRNA-A** (0.589 kb)<br>primers: pFC334-F1/*nat1*-tRNA-PS1R<br>template: pFC902 (Nødvig et al., 2018) | ***nat1*$^{PS1}$ tRNA-B** (0.427 kb)<br>primers: *nat1*-tRNA-PS1F/pFC334-R1<br>template: pFC902 (Nødvig et al., 2018) | | *in vitro* |
| **pH-OCG<br>[pEC0459]** (9.655 kb) | **pRS426$^{\Delta NcoI}$**<br>(Schumacher, 2012)<br>digested w/ *EcoR*I + *Xho*I | **P*oliC*::*mch*::T*gluc*** (2.152 kb)<br>primers: P*oliC*-pRS426-5F/T*gluc*-R1<br>template: pIGR1S-OCG [pEC0407] (unpublished) | **P*trpC*::*hph*::T*niiiA*** (2.071 kb)<br>primers: P*trpC*-SS-pRS426-3R/T*niiA*-*Pac*I-T*gluc*-F<br>template: pNAH-GGG [pEC0046] (Erdmann et al., 2022) | | *in vivo* |
| **pN-OCG<br>[pEC0460]** (9.190 kb) | **pRS426$^{\Delta NcoI}$**<br>(Schumacher, 2012)<br>digested w/ *EcoR*I + *Xho*I | **P*oliC*::*mch*::T*gluc*** (2.152 kb)<br>primers: P*oliC*-pRS426-5F/T*gluc*-R1<br>template: pIGR1S-OCG [pEC0407] (unpublished) | **P*trpC*::*nat1*::T*niiiA*** (1.598 kb)<br>primers: P*trpC*-SS-pRS426-3R/T*niiA*-*Pac*I-T*gluc*-F<br>template: pNAN-GGG [pEC0120] (Erdmann et al., 2022) | | *in vivo* |
| **pN-*capks1*-COMIL<br>[pEC0466]** (14.477 kb) | **pN-OCG** [pEC0459]<br>(this study)<br>digested w/ *Sac*II + *Not*I | ***capks1*-5'::*capks1*** (6.918 kb)<br>primers: *capks1*-pRS426-5F/*capks1*-T*gluc*-R<br>template: *C. antarcticus* gDNA | | | *in vivo* |
| **pH-*kppks1*-COMIL<br>[pEC0478]** (14.975 kb) | **pH-OCG** [pEC0460]<br>(this study)<br>digested w/ *Sac*II + *Not*I | ***kppks1* [1]** (2.562 kb)<br>primers: *kppks1*-pRS426-5F/*kppks1*-COM-5R<br>template: *K. petricola* gDNA | ***kppks1* [2]** (4.995 kb)<br>primers: *kppks1*-wtF3/*kppks1*-T*gluc*-R<br>template: *K. petricola* gDNA | | *in vivo* |

**Table S5. Transformations of *C. antarcticus* and *K. petricola* carried out in this study.**

| Strain (alias) | BAM ID | Entry | Donor DNA | CRISPR/Cas9 plasmid | Attempts | Results |
|---|---|---|---|---|---|---|
| **Δ*capks1* [N]** | **CA-0001** | *C. antarcticus* **WT:515** | **[(T*niaD*::*nat1*::P*trpC*)]** $^{\Delta capks1}$ (1.473 kb)<br>primers: *capks1*-SH5F/*capks1*-SH3R<br>template: pNDN-OGG (Schumacher, 2012) | **pAMA/tRNA-*capks1*$^{PS1}$-*capks1*$^{PS4}$**<br>[pEC0256] (this study) | Five | White colonies on transformation plates from one experiment, no growth on MEA+NTC after transfer |
| **Δ*capks1* [H]** | **CA-0002** | *C. antarcticus* **WT:515** | **[(T*niaD*::*hph*::P*trpC*)]** $^{\Delta capks1}$ (1.941 kb)<br>primers: *capks1*-SH5F/*capks1*-SH3R<br>template: pNDH-OGG (Schumacher, 2012) | **pAMA/tRNA-*capks1*$^{PS1}$-*capks1*$^{PS4}$**<br>[pEC0256] (this study) | Five | White/pinkish colonies in different numbers in four out of five attempts, grew on MEA+HYG after transfer |
| **Δ*capks1*:: *capks1* [N]** | **CA-0011** | *C. antarcticus* **Δ*pks1* [H]** | **[(*capks1*-5'::*capks1*::T*gluc*)–(T*niiA*::*nat1*::P*trpC*)]** $^{\Delta capks1}$ (8.967 kb)<br>isolated with *Swa*I from pN-*capks1*-COMiL [pEC0466] (this study) | **pAMA/tRNA-*hph*$^{PS1}$**<br>[pEC0461] (this study) | Five | No colonies on transformation plates |
| **Δ*capks1*/ Δ*caphs1-phd1* [H/N]** | **CA-0012** | *C. antarcticus* **Δ*pks1* [H]** | **[(T*niaD*::*nat1*::P*trpC*)]** $^{\Delta caphs1\text{-}phd1}$ (1.473 kb)<br>primers: *caphs1*-SH5F/*caphd1*-SH5R<br>template: pNDN-OGG (Schumacher, 2012) | **pAMA/tRNA-*caphs1*$^{PS1}$-*caphd1*$^{PS1}$**<br>[pEC0448] (this study) | Four | No colonies on transformation plates |
| **Δ*caphs1-phd1* [H]** | **CA-0017** | *C. antarcticus* **WT:515** | **[(T*niaD*::*hph*::P*trpC*)]** $^{\Delta capks1}$ (1.941 kb)<br>primers: *caphs1*-SH5F/*caphd1*-SH5R<br>template: pNDH-OGG (Schumacher, 2012) | **pAMA/tRNA-*caphs1*$^{PS1}$-*caphd1*$^{PS1}$**<br>[pEC0448] (this study) | Three | Black colonies on transformation plates from two out of three attempts, grew on MEA+HYG after transfer |
| Δ*caphs1-phd1* **[H-ONGG]** **Alias: H2B-GFP** | **CA-0021** | *C. antarcticus* **WT:515** | **[(T*niaD*::*hph*::P*trpC*)–(P*oliC*::*h2b-gfp*::T*gluc*)]** $^{\Delta caphs1\text{-}phd1}$ (4.700 kb)<br>primers: *caphs1*-SH5F/T*gluc*-*caphd1*-SH5R<br>template: pNDH-ONGG (Erdmann et al.) | **pAMA/tRNA-*caphs1*$^{PS1}$-*caphd1*$^{PS1}$**<br>[pEC0448] (this study) | Two | Black colonies on transformation plates from one experiment, grew on MEA+HYG after transfer |
| **Δ*kppks1*:: *kppks1* [H]** **Alias: Δ*kppks1*$^{COMiL}$** | **KP-0505** | *K. petricola* **Δ*pks1* [N]** | **[(*kppks1*-5'::*kppks1*::T*gluc*)–(T*niiA*:: *hph*::P*trpC*)]** $^{\Delta kppks1}$ (9.525 kb)<br>primers: *kppks1*-pRS426-5F/P*trpC*-SS-pRS426-3R; template: pH-*kppks1*$^{COMiL}$ [pEC0478] (this study) | **pAMA/tRNA-*nat1*$^{PS1}$** [pEC0462] (this study) | One | Black colonies (restored melanin synthesis) on transformation plates, grew on MEA+HYG after transfer |

## SUPPLEMENTARY REFERENCES

Erdmann, E.A., Nitsche, S., Gorbushina, A.A., and Schumacher, J. (2022) Genetic engineering of the rock inhabitant *Knufia petricola* provides insight into the biology of extremotolerant black fungi. *Front Fungal Biol* **3**: 862429.

Mumberg, D., Müller, R., and Funk, M. (1994) Regulatable promoters of *Saccharomyces cerevisiae*: comparison of transcriptional activity and their use for heterologous expression. *Nucleic Acids Res* **22**: 5767.

Noack-Schönmann, S., Bus, T., Banasiak, R., Knabe, N., Broughton, W.J., Den Dulk-Ras, H. et al. (2014) Genetic transformation of *Knufia petricola* A95 - a model organism for biofilm-material interactions. *AMB Express* **4**: 80.

Nødvig, C.S., Nielsen, J.B., Kogle, M.E., and Mortensen, U.H. (2015) A CRISPR-Cas9 system for genetic engineering of filamentous fungi. *PLoS One* **10**: e0133085.

Nødvig, C.S., Hoof, J.B., Kogle, M.E., Jarczynska, Z.D., Lehmbeck, J., Klitgaard, D.K., and Mortensen, U.H. (2018) Efficient oligo nucleotide mediated CRISPR-Cas9 gene editing in Aspergilli. *Fungal Genet Biol* **115**: 78-89.

Schumacher, J. (2012) Tools for *Botrytis cinerea*: new expression vectors make the gray mold fungus more accessible to cell biology approaches. *Fungal Genet Biol* **49**: 483-497.

Voigt, O., Knabe, N., Nitsche, S., Erdmann, E.A., Schumacher, J., and Gorbushina, A.A. (2020) An advanced genetic toolkit for exploring the biology of the rock-inhabiting black fungus *Knufia petricola*. *Sci Rep* **10**.
